# Targeting the ODC1-YBX1 axis reverses gastric cancer chemoresistance via transcriptional control of SLC7A11-mediated ferroptosis

**DOI:** 10.1038/s41420-026-03067-1

**Published:** 2026-04-14

**Authors:** Ruiqi Li, Shantanu Baral, Fanyu Zhao, Chenkai Zhang, Jiajie Zhou, Ben Li, Yifan Cheng, Dengyang Fang, Zijie Xu, Yayan Fu, Jianyue Ding, Zhen Tian, Shuai Zhao, Jie Wang, Mengli Zi, Longhe Sun, Xuetong Jiang, Qiannan Sun, Daorong Wang

**Affiliations:** 1 Northern Jiangsu People’s Hospital, Clinical Teaching Hospital of Medical School, Nanjing University, Yangzhou, China; 2 Medical College of Yangzhou University, Yangzhou, China; 3 Northern Jiangsu People’s Hospital, Yangzhou, China; 4 Yangzhou Key Laboratory of Basic and Clinical Transformation of Digestive and Metabolic Diseases, Yangzhou, China; 5 The YangZhou Clinical College of Xuzhou Medical University, Xuzhou, China

**Keywords:** Gastric cancer, Chemotherapy

## Abstract

Gastric adenocarcinoma (STAD), a leading cause of cancer mortality, faces major therapeutic challenges due to intrinsic and acquired chemoresistance. Chemoresistance is intricately linked to ferroptosis.Elucidating the mechanisms of chemotherapy resistance in STAD represents a critical unmet need to improve patient survival. This study identifies ODC1 as a crucial driver of 5-Fu resistance and suppressor of ferroptosis in STAD. Multi-dataset analysis revealed significant ODC1 overexpression in STAD tissues, correlating with advanced stage and poor survival. Functionally, ODC1 depletion inhibited proliferation, migration, invasion, and tumor growth in vitro and in vivo, while its overexpression exacerbated malignant phenotypes. Critically, ODC1 was upregulated in 5-Fu-resistant cell models, and its knockdown restored chemosensitivity by triggering ferroptosis—an iron-dependent cell death characterized by lipid peroxidation, glutathione depletion, and malondialdehyde accumulation. Mechanistically, ODC1 interacts with transcription factor YBX1 through its PLPDE_III_ODC domain. This complex binds the promoter of SLC7A11, enhancing its transcription. YBX1 silencing phenocopied ODC1 knockdown, increasing ferroptosis susceptibility; conversely, SLC7A11 overexpression or GPX4 activation (via ML334) reversed ferroptosis induced by ODC1/YBX1 inhibition. Significantly, Erastin—a SLC7A11 inhibitor—overcame YBX1-mediated resistance, synergizing with 5-Fu to induce ferroptosis and suppress tumor growth. Collectively, we unveil the ODC1-YBX1-SLC7A11-ferroptosis axis as a central mechanism of chemoresistance in STAD. Targeting this axis—via ODC1 inhibition or ferroptosis induction—represents a novel therapeutic strategy to reverse treatment resistance in gastric adenocarcinoma.

## Introduction

Gastric cancer is the fifth most commonly diagnosed cancer and the fourth leading cause of cancer-related mortality worldwide (GLOBOCAN 2020) [1]. Although incidence has declined in most developed regions, the disease burden remains substantial, with high prevalence rates persisting in East Asia, Eastern Europe, and Central America [2]. The generally poor prognosis of gastric cancer is largely attributable to the aggressive behavior of its most common pathological subtype—adenocarcinoma, particularly signet-ring cell carcinoma—which is marked by invasive growth, a tendency for asymptomatic progression in early stages, and a high potential for metastasis [3]. As a result, the majority of patients are diagnosed at advanced stages. Current treatment follows a stage-based approach: resectable disease is managed with surgery combined with perioperative chemotherapy as the standard of care, significantly improving survival; in contrast, advanced disease requires systemic chemotherapy-based combination regimens [4, 5]. Recent research has concentrated on optimizing synergistic chemotherapy regimens, with paradigm-shifting progress in molecular-targeted therapies and immune checkpoint inhibitors reshaping treatment frameworks [6].As a backbone of systemic chemotherapy, 5-fluorouracil (5-Fu) exerts broad-spectrum antitumor activity primarily through the inhibition of DNA and RNA synthesis, though it is associated with considerable toxicity [7]. A major clinical concern is that nearly half of all patients develop either primary or acquired resistance to 5-Fu, which significantly shortens overall survival [8]. Consequently, disease progression following resistance—manifesting as local recurrence or distant metastasis—requires timely therapeutic adjustment. To address these critical challenges, there is an urgent need to develop novel treatment modalities, underpinned by a deeper understanding of the molecular mechanisms driving gastric adenocarcinoma progression and drug resistance. In parallel, investigating genes implicated in gastric carcinogenesis and clarifying their functions in targeted therapeutic strategies remain essential.

Ornithine decarboxylase 1 (ODC1), the key rate-limiting enzyme in polyamine biosynthesis, catalyzes the conversion of ornithine to putrescine, thereby directly regulating intracellular homeostasis of bioactive polyamines including spermidine and spermine [9]. Notably, dysregulated accumulation of intracellular polyamines is closely associated with the development and progression of multiple malignancies [10]. In neuroblastoma, ODC1 knockdown has been shown to suppress cancer cell proliferation, and polyamine-targeting antagonists have demonstrated clinical efficacy in delaying disease progression [11]. Similarly, in hepatocellular carcinoma (HCC), ODC1 expression is significantly upregulated, and its silencing effectively inhibits cancer cell proliferation, migration, and invasion, while inducing cell cycle arrest at the G1/S phase [12]. However, in gastric adenocarcinoma, the relationship between ODC1 expression patterns and clinical stages remains unclear. Moreover, the specific mechanisms by which ODC1 influences malignant progression—particularly those involving ferroptosis—await systematic investigation.

Ferroptosis is an iron-dependent form of regulated cell death driven by uncontrolled lipid peroxidation within the plasma membrane or organelle membranes [13]. It is distinguished from other cell death modalities by unique morphological features—such as mitochondrial shrinkage with diminished cristae and increased membrane density—and biochemical hallmarks including malondialdehyde accumulation and glutathione depletion [14]. Mechanistically, ferroptosis is widely understood as a consequence of dysregulated cellular metabolism and redox imbalance, leading to lethal lipid peroxidation [15]. In iron-rich microenvironments, excess hydrogen peroxide (H₂O₂) can react with Fe²⁺ via the Fenton reaction, generating highly reactive hydroxyl radicals (OH) [16]. These radicals initiate the peroxidation of polyunsaturated fatty acid (PUFA)-containing phospholipids at bis-allylic sites, resulting in membrane destabilization and eventual rupture [17]. Accumulating evidence underscores ferroptosis as a critical factor in tumor biology and treatment. Multiple conventional therapies—including radiotherapy, chemotherapy, and immunotherapy—can induce ferroptosis, contributing to their antitumor efficacy [18–20]. Molecularly, ferroptosis may interact with oncogenic signaling pathways such as the WNT/MYC axis, forming positive feedback loops that amplify ferroptotic effects and sensitize neighboring cells, thereby promoting its propagation throughout tumor regions [21]. Conversely, cancer cells can develop resistance to ferroptosis through oncogene-driven signaling or metabolic adaptations, establishing it as a natural barrier against tumor progression [22, 23]. Notably, ferroptosis induction has been reported to enhance tumor sensitivity to chemotherapy, enabling synergistic effects between ferroptosis inducers and chemotherapeutic agents while reducing systemic toxicity and overcoming drug resistance [24, 25]. However, it remains unclear whether targeting specific genes associated with gastric adenocarcinoma can chemosensitize tumors through ferroptosis induction, thereby improving therapeutic outcomes.

In this study, we demonstrate that ODC1 interacts with the transcription factor YBX1, facilitating its recruitment to the SLC7A11 promoter and enhancing the transcription of this key ferroptosis suppressor. Both in vitro and in vivo, ODC1 depletion promoted ferroptosis and restored chemosensitivity in gastric adenocarcinoma cells. Our findings establish a crucial mechanism whereby ODC1 confers 5-Fu resistance in gastric adenocarcinoma by inhibiting ferroptosis, positioning ODC1 targeting as a promising therapeutic strategy to counteract chemotherapy resistance in STAD through ferroptosis induction.

## Result

### ODC1 is upregulated in STAD and predicts poor prognosis

**Fig. 1:**
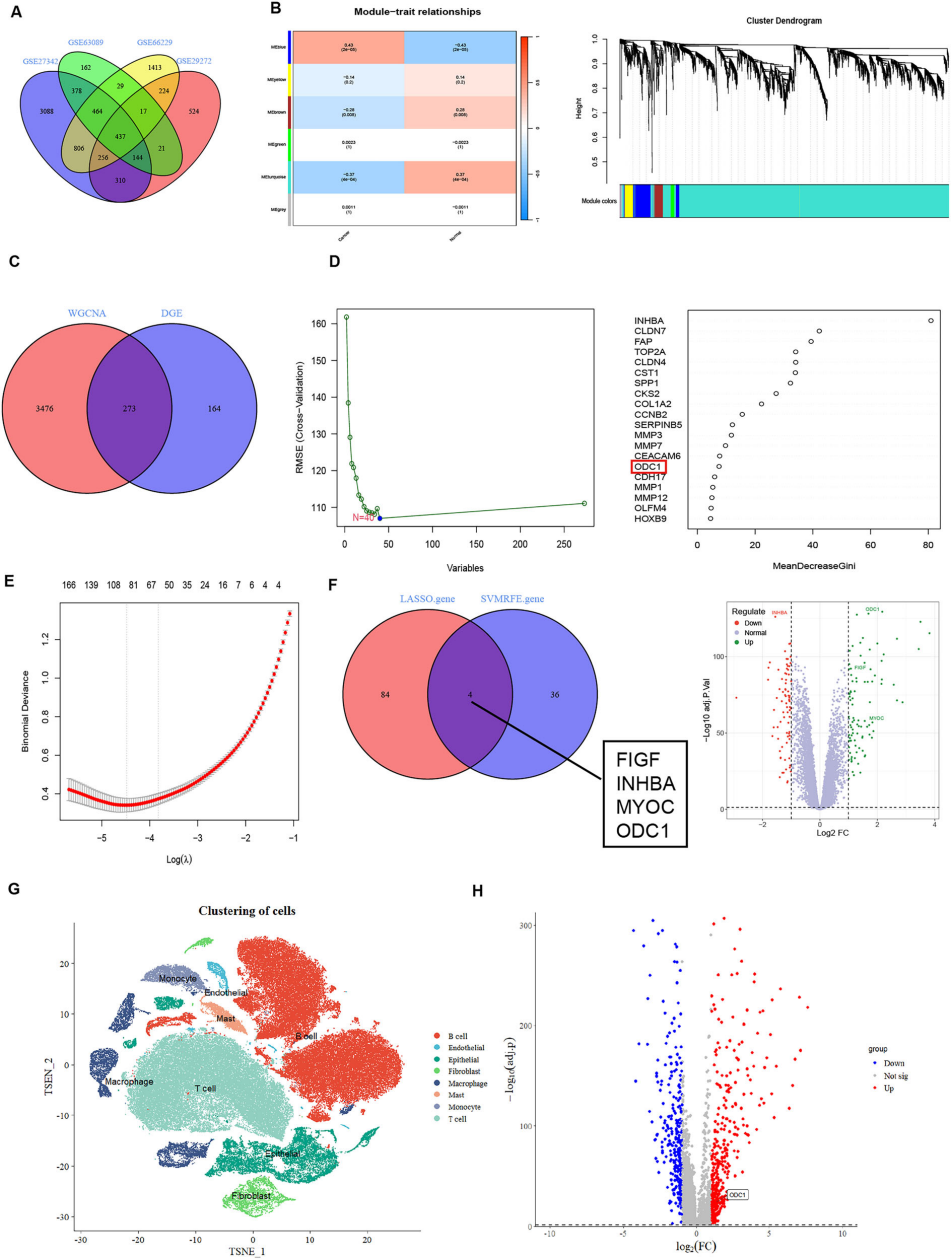
Multi-dataset integration and machine learning for identifying STAD hub genes. **A** Venn diagram of differentially expressed genes (DEGs) in four STAD transcriptome datasets (GSE27342, GSE63089, GSE66229, GSE29272), identifying 437 consistently dysregulated genes (tumor vs. adjacent tissues across all cohorts). **B** Module-trait relationships in WGCNA revealing MEblue module with strongest phenotype correlation (r = 0.43, P = 2×10 − 5).Sample dendrogram and trait heatmap after multi-batch integration. **C** Overlap of DEGs (437 genes) and MEblue module genes (3,749 genes) yielding 273 candidate STAD-associated genes. **D** The reliable SVM-based predictive model, with low RMSE, identified the top 20 contributing genes, including ODC1 (highlighted in red). **E** LASSO regression coefficient profiles showing feature selection shrinkage. Vertical line indicates optimal lambda. **F** The intersection of genes obtained from the two machine learning methods was taken, and the expression levels of these four genes were displayed in a volcano plot. **G** t-SNE plot of 116,981 single cells from GSE201347, annotated to 8 major lineages (Epithelial cells: red). **H** Violin plots showing significant ODC1 upregulation in epithelial cells (log₂FC = 1.83, P = 4.2×10-15, Wilcoxon test).

To elucidate the role of ODC1 in STAD, we analyzed four STAD-related RNA transcriptome sequencing (RNA-seq) datasets from the GEO database: GSE27342 (University of Georgia, 160 samples), GSE63089 (Jilin University, 90 samples), GSE66229 (Merck & Co, 400 samples), and GSE29272 (National Cancer Institute, USA, 268 samples). First, we intersected differentially expressed genes (DEGs) between tumor and adjacent tissues across all four datasets, identifying 437 consistently dysregulated genes (Fig. 1A). After batch-effect correction, we integrated the datasets and constructed a gene co-expression network using weighted gene co-expression network analysis (WGCNA) to identify functional modules. The “MEblue” module showed the highest correlation with phenotype (r = 0.43, P = 2e-5) and was selected for further analysis (Fig. 1B), containing 3749 genes. Intersecting genes from both screening methods yielded 273 overlapping genes (Fig. 1C). To identify core genes likely involved in STAD pathogenesis, we applied two machine learning algorithms—support vector machine (SVM) (Fig. 1D) and least absolute shrinkage and selection operator (LASSO) (Fig. 1E)—to these overlapping genes, ultimately identifying four core candidates: FIGF, INHBA, MYOC, and ODC1(Fig. 1F).

Subsequently, we integrated the four GEO datasets as a training set and evaluated the diagnostic efficacy of these genes using receiver operating characteristic (ROC) curves. ODC1 demonstrated the highest diagnostic value for STAD, with an area under the curve (AUC) of 0.923 (Supplementary Fig. S1A). To validate this, we analyzed three additional GEO datasets (GSE13911, Merck & Co., 69 samples; GSE79973, Zhejiang Provincial People’s Hospital, 20 samples; GSE220917, Heidelberg University, 23 samples) as an independent validation set. After batch-effect correction, ROC analysis again confirmed ODC1’s superior diagnostic accuracy (AUC = 0.890, Supplementary Fig. S1B). Consistently, ODC1 expression was significantly elevated in tumor tissues versus adjacent tissues across all seven GEO datasets (P < 0.05, Supplementary Fig. S1C). To validate the specific expression level of ODC1 in human gastric cancer specimens, we performed single-cell RNA sequencing analysis on 22 samples (12 gastric tumor and 10 non-tumor samples) from the GEO dataset GSE201347. After filtering out cells with low RNA abundance and high mitochondrial RNA content, a total of 116,981 cells were retained for subsequent analysis. t-SNE visualization identified 21 cell clusters at a resolution of 0.5. Based on established marker expression, we ultimately annotated 8 distinct cell types, including 11,408 epithelial cells. Differential gene analysis of epithelial cell populations between tumor and non-tumor groups using FindMarkers revealed significant upregulation of ODC1 in the tumor group (Fig. 1G, H).We further investigated ODC1’s expression and clinical impact using TCGA data, confirming its upregulation in STAD tissues (Supplementary Fig. S1F). High ODC1 expression correlated with advanced clinical stages (T-stage and N-stage, Supplementary Fig. S1D, E) and significantly reduced 10-year survival (Supplementary Fig. S1G), supporting its role as a prognostic biomarker.

**Fig. 2:**
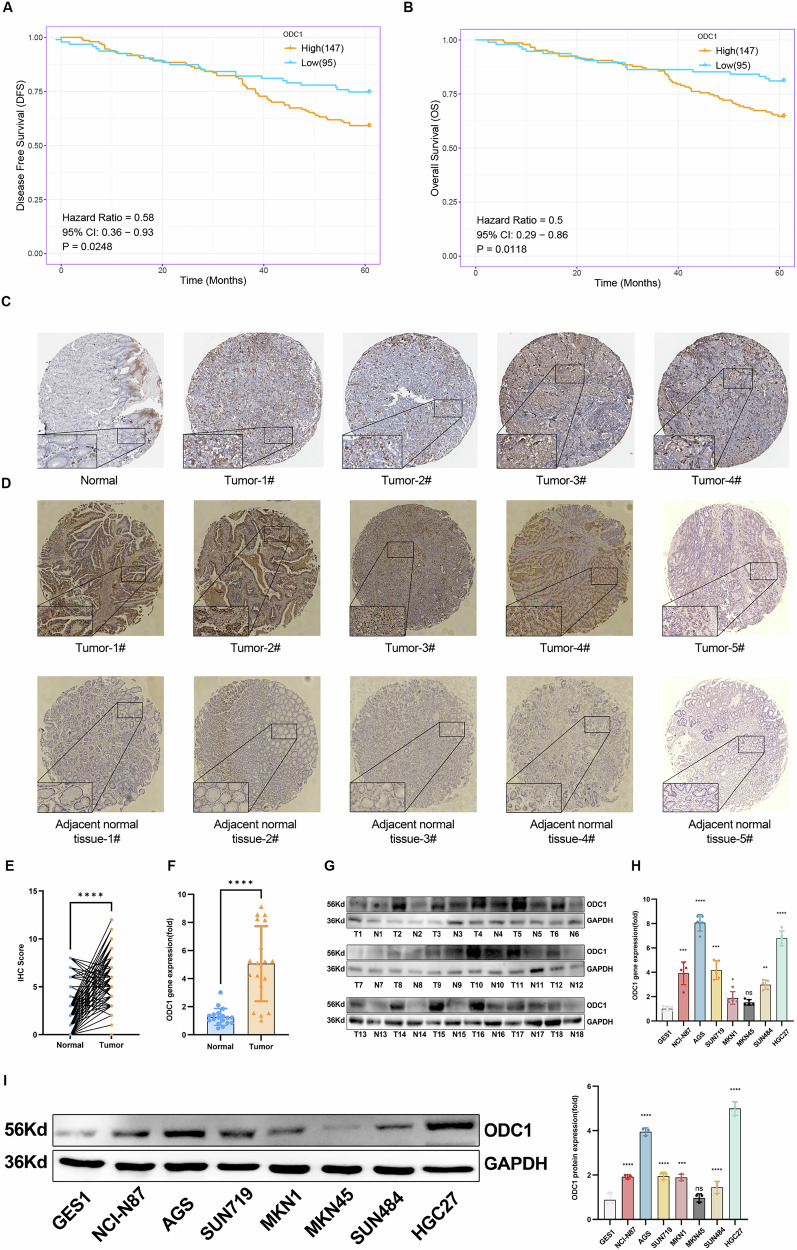
Clinical and molecular validation of ODC1 as a STAD oncoprotein. **A, B** Kaplan-Meier analysis of 5-year Disease-free survival (DFS) and overall survival (OS) of 242 STAD patients in our center stratified by ODC1 expression. While curves showing high ODC1 group had shorter progression-free intervals. **C** HPA database reference confirming ODC1 protein overexpression in STAD tissues. **D**, **E** Immunohistochemical (IHC) analysis performed on a tissue microarray constructed from surgical specimens at our center confirmed that ODC1 is highly expressed in gastric cancer tissues by Semi-quantitative H-score analysis. **F** RT-qPCR of ODC1 mRNA in 18 paired clinical specimens. **G** Western blot quantification showing ODC1 increase in tumors. **H** RT-qPCR analysis across gastric cell lines confirming cancer cell-specific ODC1 elevation (vs. GES-1). **I** ODC1 protein expression levels in seven STAD cell lines and non-tumor cell line GES-1. Data are marked as the means ± SD. *P < 0.05, **P < 0.01, ***P < 0.001,****P < 0.0001.

To validate these bioinformatic findings, we conducted a 60-month overall survival (OS) follow-up of 242 STAD patients from our center. After excluding non-disease-related deaths, disease-free survival (DFS) analysis confirmed that high ODC1 expression predicted significantly poorer prognosis (Fig. 2A, B; Supplementary Table 1). Immunohistochemical (IHC) staining of tissue microarrays revealed stronger ODC1 expression in STAD tissues than in adjacent tissues, consistent with the Human Protein Atlas (HPA) database (Fig. 2C-E). Additionally, quantitative reverse transcription PCR (RT-qPCR) and Western blot (WB) analyses of 18 paired STAD and adjacent tissues confirmed ODC1 upregulation at both mRNA and protein levels (Fig. 2F, G). We further validated this in normal gastric mucosal cell line GES1 and seven gastric cancer cell lines, where ODC1 expression was consistently elevated in cancer cells (Fig. 2H, I).These results suggest that ODC1 is upregulated in STAD.

### ODC1 Enhances the Proliferation, Migration, and Invasion of STAD Cells in Vitro or in Vivo

Building upon the differential basal expression profiles of ODC1 across gastric cancer cell lines established in prior analyses, we strategically selected AGS and HGC27 cells (exhibiting relative high endogenous ODC1 expression) for lentiviral-mediated ODC1 knockdown, while utilizing MKN45 cells (characterized by relative low basal expression) for ODC1 overexpression models. Transfection efficiency was rigorously validated through RT-qPCR and WB, confirming successful genetic manipulation across all three cell lines (Supplementary Fig. S2A-F).

**Fig. 3:**
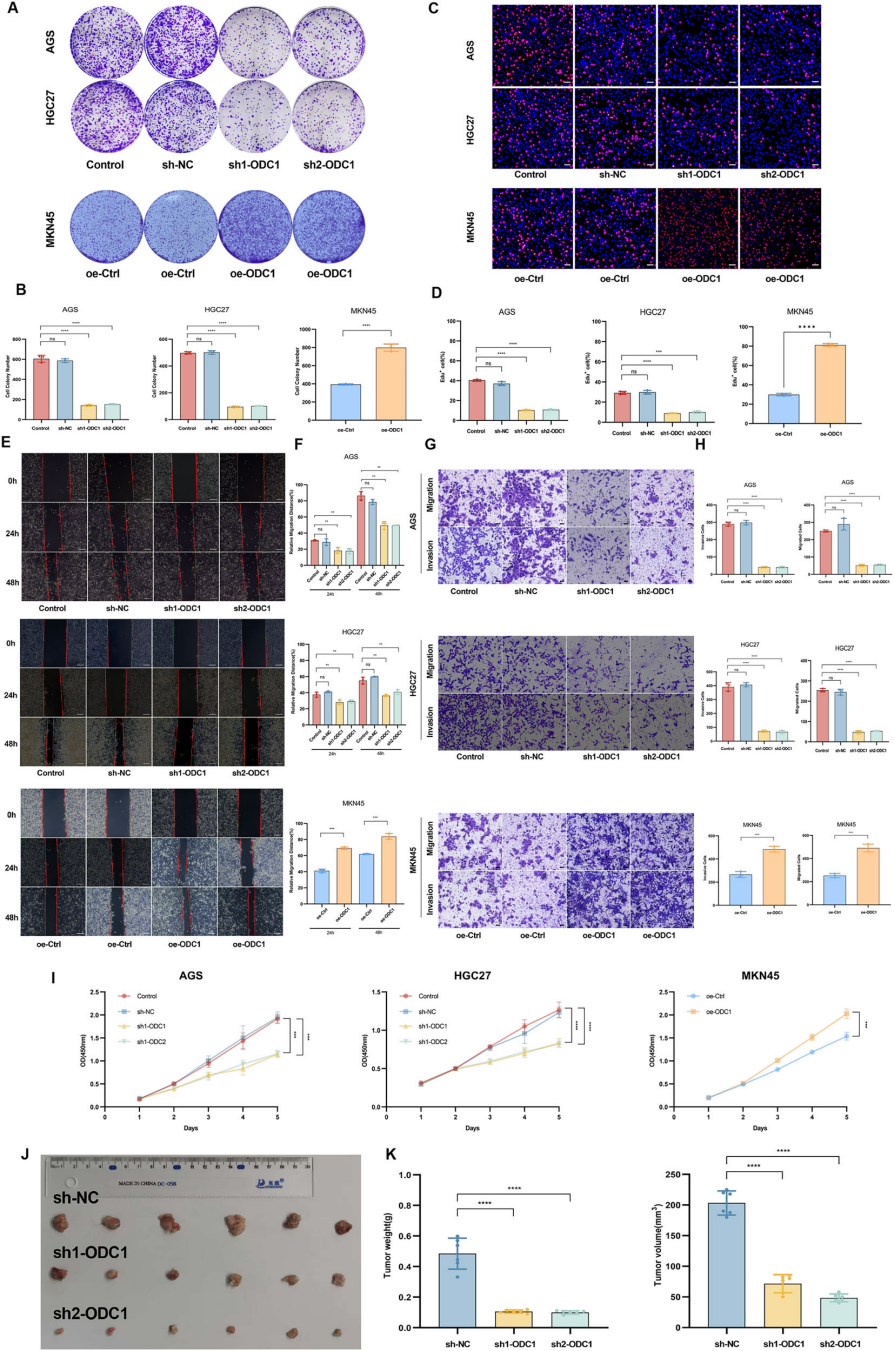
ODC1 drives malignant phenotypes via clonogenicity, proliferation and metastasis programs. **A**, **B** Colony formation assays showing ODC1 loss-of-function reduces clonogenic capacity while overexpression enhances it. **C**, **D** EdU staining confirming proliferative impairment in HGC-27 and AGS cells transfected with sh-ODC1 or MKN45 transfected with oe-ODC1.Scale bar = 20μm. **E**, **F)** Wound healing dynamics at 0/24 h/48 h: HGC-27 and AGS cells transfected with sh-ODC1 or MKN45 transfected with oe-ODC1.Scale bar = 200 μm. **G**, **H** Transwell assays showing invasive potential in HGC-27 and AGS cells transfected with sh-ODC1 or MKN45 transfected with oe-ODC1.Quantification of invaded cells per field Scale bar = 50μm. **I** CCK-8 proliferation over 120 h in HGC-27 and AGS cells transfected with sh-ODC1 or MKN45 transfected with oe-ODC1. **J** Tumor samples from the indicated groups of mice expressing the sh-NC, sh1-ODC1 or sh2-ODC1 plasmids (*n* = 6 in each). **K** Measurement data for tumor volume and weight are expressed as mean ± SD *P < 0.05, **P < 0.01, ***P < 0.001,****P < 0.0001.

Functional characterization revealed that ODC1 depletion profoundly attenuated clonogenic potential, as evidenced by a significant reduction in colony formation capacity (P < 0.01), whereas ODC1 overexpression markedly enhanced this phenotype (Fig. 3A, B). Concomitantly, CCK-8 proliferation assays and 5-ethynyl-2’-deoxyuridine (EdU) incorporation analysis demonstrated that ODC1 knockout substantially suppressed cellular proliferation kinetics, while its ectopic expression accelerated growth rates (Fig. 3C, D, I).Migration capabilities were quantitatively assessed via wound healing assays. sh-ODC1 cells exhibited significantly impaired wound closure dynamics, with statistically reduced migration rates compared to controls (P < 0.05, Fig. 3E, F). Transwell migration and matrigel invasion assays further corroborated these findings: ODC1 knockdown drastically impaired transmigration and invasive properties in AGS and HGC27 cells, whereas ODC1-overexpressing MKN45 cells displayed augmented migratory and invasive phenotypes (Fig. 3G, H).

To evaluate tumorigenic relevance in vivo, a xenograft model was generated by subcutaneous implantation of stable ODC1-knockdown HGC27 cells in immunodeficient mice. Consistent with in vitro observations, sh-ODC1 tumors demonstrated significantly diminished tumor volume and weight progression and growth kinetics relative to sh-NC controls (Fig. 3J, K). Immunohistochemical analysis of resected tumors confirmed effective ODC1 ablation in the knockdown cohort, accompanied by substantial downregulation of the proliferation marker Ki-67 (Supplementary Fig. S2G).

Collectively, these data establish ODC1 as a critical regulator of multiple malignant phenotypes—including clonogenicity, proliferative capacity, migration, invasion, and in vivo tumor growth—in gastric adenocarcinoma. This comprehensive functional validation underscores the necessity to elucidate the precise molecular mechanisms through which ODC1 orchestrates gastric carcinogenesis and progression.

### ODC1 drives acquired and intrinsic 5-Fu resistance in STAD

Building upon our previous findings implicating ODC1 in regulating malignant phenotypes of gastric cancer, we performed next-generation sequencing comparing ODC1-low and ODC1-normal expressing cell lines. Kyoto Encyclopedia of Genes and Genomes(KEGG) and Gene Set Enrichment Analysis (GSEA) revealed significant enrichment of differentially expressed genes in processing of drugs (Supplementary Fig. S3A, B), suggesting ODC1’s potential involvement in chemoresistance regulation. Given that 5-Fu serves as a cornerstone chemotherapeutic agent for advanced gastric cancer [26], we established stable 5-Fu-resistant cell models (designated RE-AGS and RE-HGC27) from AGS and HGC27 parental lines using a stepwise dose-escalation protocol.Following 6 months of induction and 2-week drug withdrawal validation, resistant cells exhibiting resistance indices (RI) > 2 were obtained (Supplementary Fig. S3C), with resistance stability confirmed through half-maximal inhibitory concentration (IC50) determination(AGS_IC50_ = 11.84uM VS. Re-AGS_IC50_ = 138.50uM; HGC27_IC50_ = 11.21uM VS. Re-HGC27_IC50_ = 84.33uM). Consistent with reports that chemoresistant cells enter a slow-cycling, dormancy-like state to maintain genomic stability [27]. We observed downregulation of proliferation and cell cycle regulators such as PCNA, cyclin D1, CDK4 and CDK6(Supplementary Fig. S3D). Flow cytometric analysis revealed pronounced G1-phase arrest with concomitant reduction in S and G2/M phase populations in resistant versus parental cells (Supplementary Fig. S3E). Despite their slower proliferation, resistant lines demonstrated significantly enhanced clonogenic survival and viability following 5-Fu exposure in colony formation assays (Supplementary Fig. S3F), further validated by increased Calcein-AM/PI viable cell populations via dual-fluorescence staining (Supplementary Fig. S3G).

**Fig. 4:**
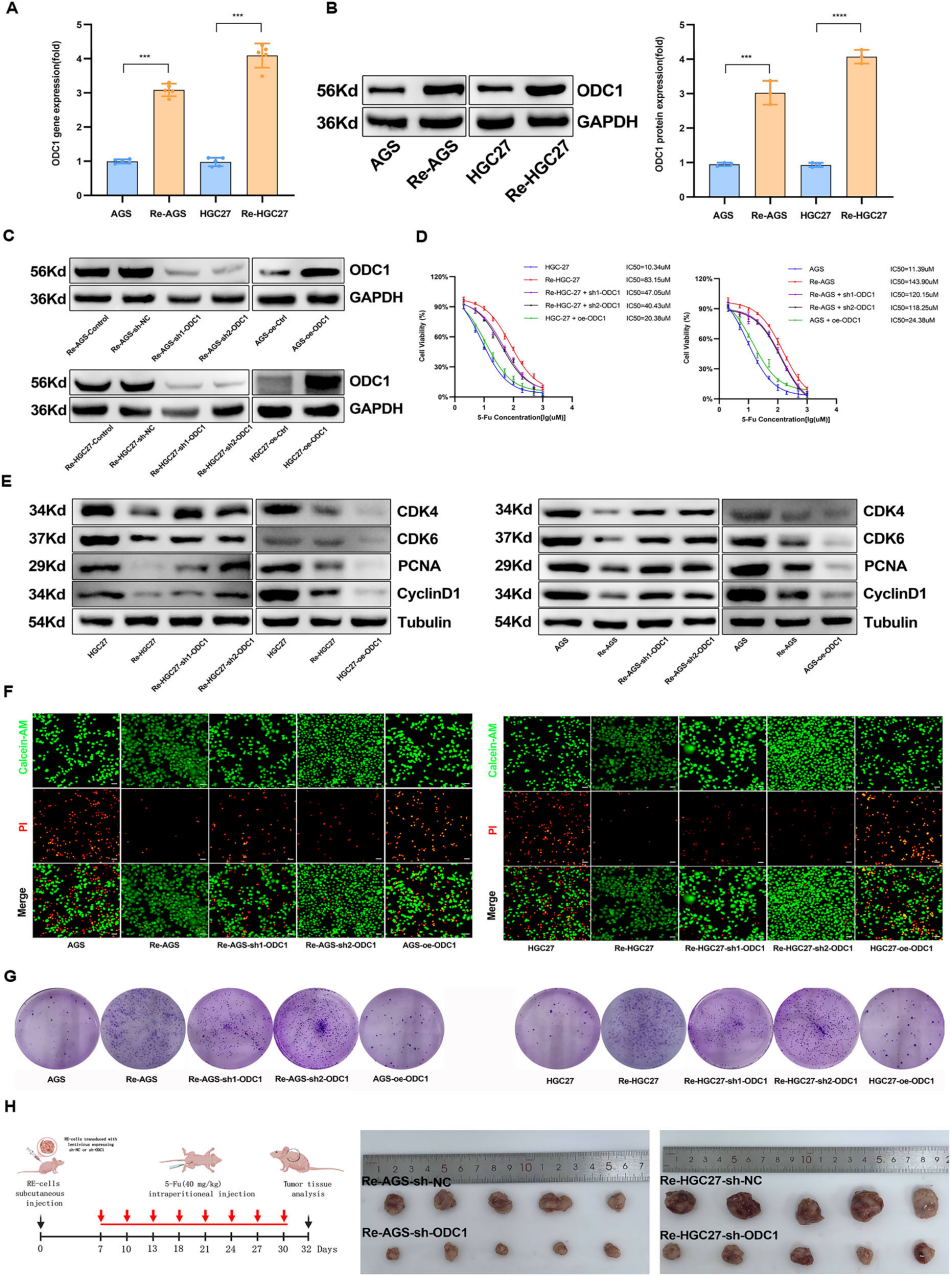
ODC1 functionally drives 5-Fu chemoresistance through dormancy reprogramming. **A**, **B** Western blot and RT-qPCR validation showing increased ODC1 expression in 5-Fu-resistant lines. **C** ODC1 knockdown in resistant lines and overexpression (oe-ODC1) in parental cells confirmed by Western Blot. **D** The construction of the drug-resistant cell model(Knockdown of ODC1 in drug-resistant cells and overexpression of ODC1 in parental cells.) was validated by calculating the IC50 through a concentration-gradient assay. **E** The expression levels of cell cycle proteins were verified by Western blot in genetically edited drug-resistant cells and parental cells. **F** Calcein-AM/PI dual staining post-5-Fu, showing enhanced viable cell populations in genetically edited drug-resistant cells and parental cells.scale bars=20μm. **G** Colony formation assays showing ODC1 loss-of-function reduces resistance capacity in genetically edited drug-resistant cells and parental cells. **H** Representative allograft tumors following subcutaneous injection of ODC1 knockdown and sh-NC in Re-AGS and Re-HGC27 cells and treatment with 5-Fu (40 g/kg).*P < 0.05, **P < 0.01, *** *P* < 0.001,****P < 0.0001.

Notably, ODC1 expression was markedly upregulated in resistant cells (Fig. 4A, B). To establish causality, we performed ODC1 knockdown in resistant lines and overexpression in parental cells (Fig. 4C). IC50 assessment demonstrated that ODC1 knockdown reduced chemoresistance in resistant cells, while ODC1 overexpression enhanced resistance in parental lines (Fig. 4D). WB analysis of cell cycle/proliferation markers revealed that ODC1 depletion reversed the dormancy-like phenotype in resistant cells, whereas ODC1 overexpression suppressed proliferative activity in parental cells post-chemotherapy (Fig. 4E). Calcein-AM/PI staining confirmed increased drug-induced cytotoxicity in ODC1-knockdown resistant cells and acquired resistance in ODC1-overexpressing parental cells (Fig. 4F), findings corroborated by clonogenic assays (Fig. 4G).

In xenograft models using 4-week-old nude mice implanted with ReHGC27-shODC1 or ReAGS-shODC1 cells, biweekly 5-Fu administration (40 μg/g) for 8 cycles resulted in significantly reduced tumor volumes compared to controls, demonstrating that ODC1 ablation reverses chemoresistance in vivo (Fig. 4H). Collectively, these results demonstrate that ODC1 upregulation confers 5-Fu resistance in STAD by promoting a dormancy-like state and cell cycle arrest, establishing ODC1 as a key mediator of chemoresistance in both de novo and acquired resistance models.

### ODC1 confers 5-Fu resistance by suppressing ferroptosis

To elucidate the mechanism by which ODC1 confers chemoresistance, we conducted controlled experiments in both cell line models. Under basal conditions, high-dose 5-Fu treatment induced significant cell death characterized by elevated cleaved-caspase 3 (apoptosis marker) and PGAM5 (necroptosis marker), alongside reduced expression of GPX4—a phospholipid hydroperoxidase that protects against membrane lipid peroxidation and ferroptosis. Notably, upon ODC1 depletion under continued 5-Fu exposure, cleaved-caspase 3 and PGAM5 levels showed no further increase, whereas GPX4 expression decreased more substantially (Supplementary Fig. S4A), suggesting ferroptosis may contribute to ODC1-modulated cell death. Supporting this, bioinformatic analysis of STAD from TCGA revealed significant correlations between ODC1 expression and key ferroptosis regulators such as GPX4, SLC7A11, ACSL4, AKR1B1(Supplementary Fig. S4B) as reported [28–31]. To functionally validate ferroptosis involvement, we employed specific inhibitors: ferrostatin-1 (Fer-1) for ferroptosis, necrosulfonamide (NEC) for necroptosis, and zVAD-fmk (Z-V) for apoptosis. Each inhibitor selectively blocked its respective cell death pathway. We then modulated ODC1 expression in resistant cells and assessed 5-Fu-induced cytotoxicity with or without Fer-1. Calcein-AM/PI dual-staining demonstrated that ODC1 knockdown sensitized cells to 5-Fu, markedly reducing viability and increasing cell death. Crucially, pretreatment with Fer-1—but not NEC or Z-V—rescued the viability loss and cell death enhancement in ODC1-depleted cells (Supplementary Fig. S4C). The reduction or loss of mitochondrial cristae accompanied by increased membrane density is a classic morphological feature of ferroptosis. Accordingly, upon 5-Fu stimulation, drug-resistant cells observed under electron microscopy exhibited characteristic mitochondrial alterations of ferroptosis, which included fewer and smaller cristae, as well as an increase in mitochondrial membrane density.We examined six fields of view for each group. In both cell lines Re-AGS and Re-HGC27, the proportion of mitochondria exhibiting shrinkage and cristae loss was significantly increased in the 5-Fu-treated groups (by an average of 42% and 48%, respectively), further suggesting that the chemotherapeutic agent induces cell death through the ferroptosis pathway (Supplementary Fig. S4G). These findings were corroborated by CCK-8 assays (Supplementary Fig. S4D), collectively establishing ferroptosis as the primary mechanism through which ODC1 regulates chemoresistance.

**Fig. 5:**
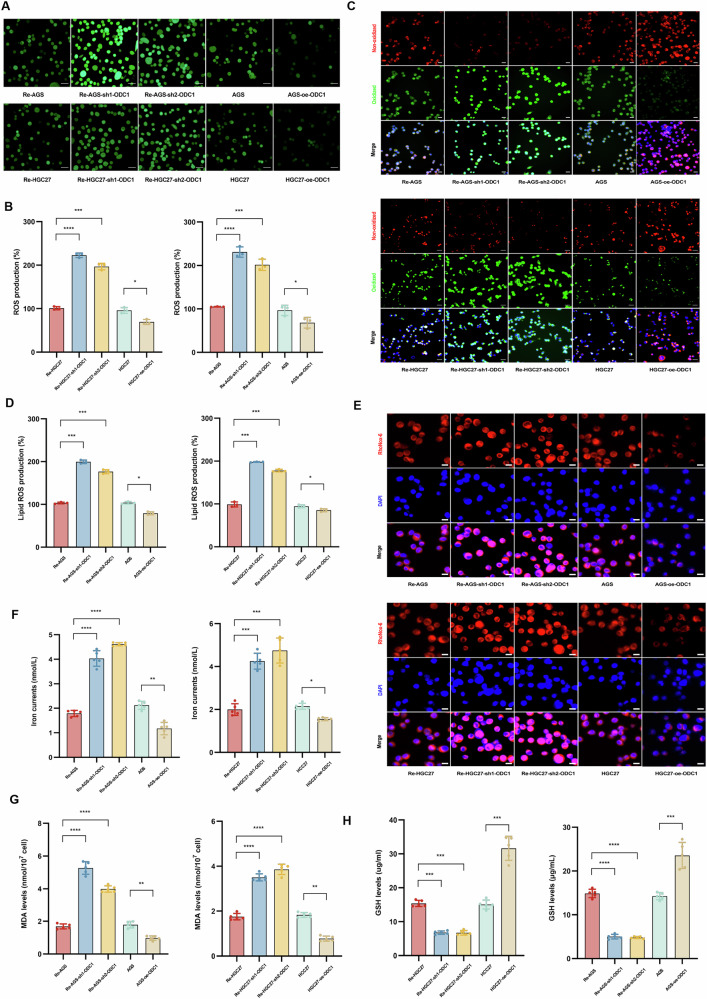
ODC1 confers chemoresistance through bidirectional regulation of ferroptosis. **A, B** ROS production was measured by DCF-DA staining in genetically edited drug-resistant cells and parental cells.scale bars=20 μm. **C, D** Lipid ROS generation in genetically edited drug-resistant cells and parental cells was measured by C11-BODIPY581/591 staining, scale bars = 20 μm. **E, F** Iron dysregulation detected by RhoNox-6 fluorescence and Ferrous iron assay kit.scale bars = 20 μm. **G** MDA levels by MDA assay kit. **H** GSH contents by GSH assay kit.*P < 0.05, **P < 0.01, *** P < 0.001,****P < 0.0001.

Subsequent investigation focused on ferroptosis-related biomarkers and phenotypes using two independent experimental sets to preclude 5-Fu-induced bias. Under drug-free conditions, analysis of five established cell lines revealed that ODC1 knockdown downregulated ferroptosis suppressors GPX4 and SLC7A11 while upregulating pro-ferroptotic regulators ACSL4 and AKR1B1, a pattern confirmed by GPX4 immunofluorescence indicating ODC1 actively inhibits ferroptosis execution (Supplementary Fig. S4E, F). Phenotypic characterization demonstrated ODC1 depletion elevated reactive oxygen species accumulation (H₂DCFDA assay; Fig. 5A, B), increased lipid peroxidation evidenced by BODIPY™ 581/591 C11 fluorescence shift (Fig. 5C, D), reduced glutathione (GSH) levels, and elevated malondialdehyde (MDA) production (Fig. 5G, H). Concurrently, direct iron quantification assays and RhoNox-6 fluorescent probing confirmed significantly augmented intra- and extracellular iron levels following ODC1 knockdown (Fig. 5E, F), collectively validating enhanced ferroptosis. These manifestations were consistently recapitulated under 5-Fu treatment, with ODC1 knockdown upregulating pro-ferroptotic proteins (Supplementary Fig. S5A, B), amplifying lipid radical formation (Supplementary Fig. S5C-F), and exacerbating biochemical alterations (Supplementary Fig. S5G-J). Notably, only the iron chelator deferoxamine (DFO) – which blocks iron-dependent Fenton reactions – reversed erastin-induced ODC1 upregulation, whereas the lipid peroxide scavenger ferrostatin-1 (Fer-1) showed no such effect (Supplementary Fig. S5K). This mechanistic distinction establishes a bidirectional regulatory relationship wherein ODC1 deficiency triggers iron-driven ferroptosis execution, while ferroptotic stimuli conversely modulate ODC1 expression through DFO-sensitive iron signaling pathways.

### ODC1 and YBX1 co-regulate SLC7A11 expression through protein interaction

**Fig. 6:**
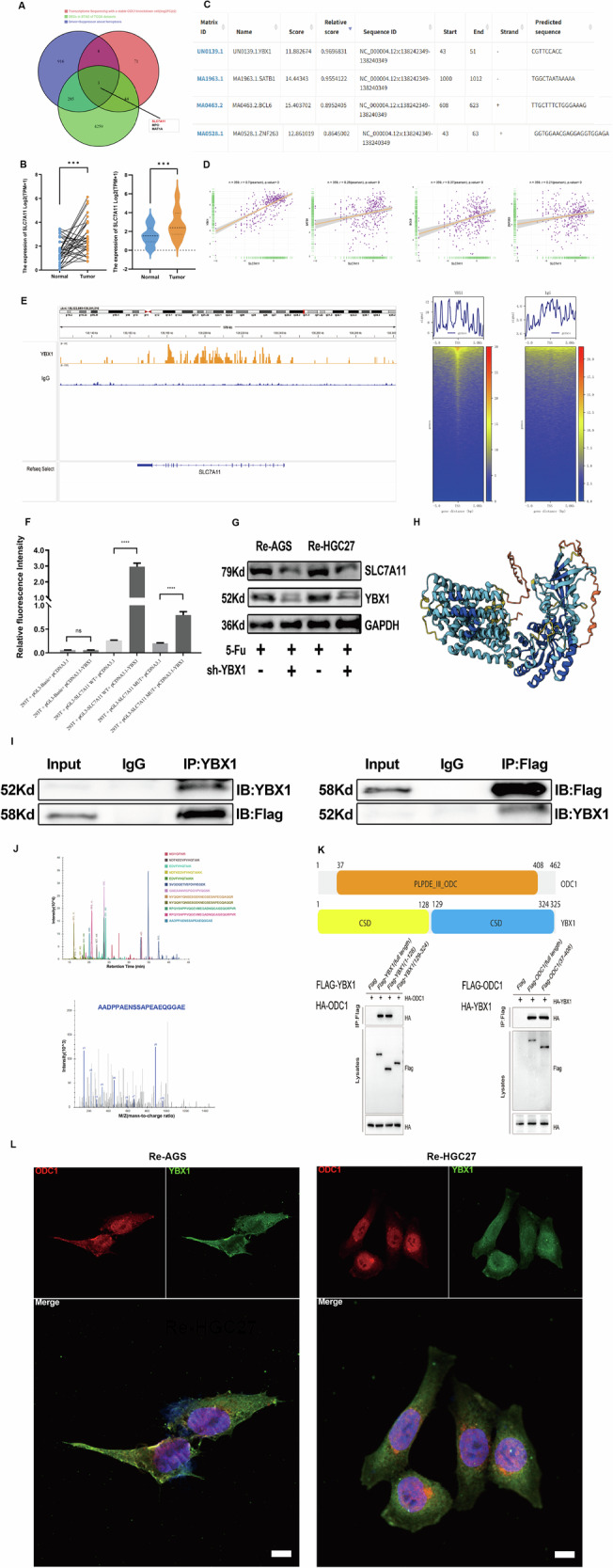
ODC1 recruits YBX1 to transactivate SLC7A11 and suppress ferroptosis. **A** Venn intersection of ODC1-KD transcriptome (|log₂FC | ≥ 2),FerrDbV2 ferroptosis genes (*n* = 1210) and TCGA-STAD DEGs (*n* = 4591). **B** The expression level of SLC7A11 in the TCGA gastric cancer database. **C** The transcription factor with the highest SLC7A11 score predicted by JASPAR. **D** Correlation analysis between the four transcription factors and SLC7A11 in the TCGA database. **E** CUT&Tag reveals YBX1 enrichment at SLC7A11 locus and related Heatmap shows locus-specific binding. **F** Luciferase assay confirms YBX1 drives SLC7A11 transcription. **G** Western blot under 5-Fu shows shYBX1 ablates SLC7A11 in resistant cells. **H** AlphaFold-predicted ODC1-YBX1 complex (confidence score pLDDT=89.7). Interface involves ODC1 β-sheet/YBX1 cold shock domain. **I** Co-IP confirms ODC1 binds YBX1. **J** MS verification: TIC/EIC traces identify YBX1 peptide AADPPAENSSAPEAEQGGAE. **K** The COIP assay further elucidated the specific protein domains involved in the molecular interaction between YBX1 and ODC1. **L** IF images of Re-AGS and Re-HGC27 cells co-transfected of ODC1 and YBX1. Scale bar = 15 μm. *P < 0.05, **P < 0.01, *** P < 0.001,****P < 0.0001.

Given the inherent complexity linking ferroptosis and drug resistance mechanisms—which cannot be attributed to isolated genetic alterations—we sought to delineate the specific pathways through which ODC1 modulates ferroptosis. Transcriptomic analysis was performed using a stringent cutoff of |log2FC | ≥ 2 to compensate for statistical test power and ensure reliability, identifying 124 differentially expressed genes upon ODC1 knockdown [32]. Concurrently, 1210 ferroptosis-related driver/suppressor genes were curated from “FerrDbV2”(http://www.zhounan.org/ferrdb/current/), a rigorously validated database supported by high-impact publications and established repositories. Intersectional analysis of these datasets with 4591 STAD-specific DEGs from TCGA revealed three overlapping genes: SLC7A11, MPO, and MAT1A. Among these, SLC7A11 exhibited the most pronounced ODC1-dependent expression changes with the highest statistical significance (Fig. 6A, B). We therefore preliminarily propose that ODC1 influences ferroptosis-mediated drug resistance primarily through SLC7A11 regulation.

While the aforementioned bioinformatic and sequencing analyses demonstrate a correlation between ODC1 and SLC7A11 at the transcriptional level, it is essential to recognize that biological phenotypes are predominantly governed by proteins—the fundamental functional units. The process of gene transcription and translation into proteins is highly complex, involving critical post-translational modifications such as ubiquitination and methylation [33]. We therefore initially hypothesized that ODC1 might directly interact with SLC7A11 at the protein level. SLC7A11, a membrane transporter responsible for glutamine synthesis and transport, serves as a key initiator of ferroptosis [34, 35]. Surprisingly, co-immunoprecipitation (Co-IP) assays conducted in three independent replicates failed to detect any positive interaction between ODC1 and SLC7A11. This unexpected result prompted us to redirect our investigation toward the alternative hypothesis that ODC1 regulates SLC7A11 at the transcriptional level.Therefore,we initiated transcription factor (TF) prediction for SLC7A11 using NCBI (https://www.ncbi.nlm.nih.gov/) and JASPAR (https://jaspar.elixir.no/). By querying the 1,000–2,000 bp upstream genomic region of SLC7A11 via NCBI, we identified four top candidate TFs—YBX1, SATB1, BCL6, and ZNF263—based on correlation coefficient scoring (Fig. 6C). Subsequent correlation analysis between SLC7A11 and these candidate TFs was performed within the STAD dataset of TCGA. Pearson correlation analysis revealed a robust positive correlation between SLC7A11 and YBX1 (r = 0.7, *p* < 0.001), providing compelling evidence that YBX1 is a putative transcriptional regulator of SLC7A11 (Fig. 6D).YBX1 is a multifunctional DNA/RNA-binding protein belonging to a large protein family characterized by an evolutionarily conserved cold shock domain [36]. It regulates diverse DNA/RNA-dependent processes, including transcription, DNA repair, mRNA stability, splicing, and translation [37, 38]. Current evidence indicates that YBX1 participates in critical cellular processes such as proliferation, differentiation, autophagy, stress response, and oncogenesis at the cellular level [39, 40]. To elucidate the regulatory mechanism of YBX1 on SLC7A11, we first employed Cleavage Under Targets & Tagmentation (CUT&Tag) technology to analyze the binding between YBX1 and the SLC7A11 genomic region.YBX1 exhibited a significant enrichment peak at the SLC7A11 locus, whereas the IgG negative control showed no detectable signal. The corresponding heatmap and signal profile further confirmed that YBX1 binding intensity in the target genomic region substantially exceeded that of IgG, indicating direct binding of YBX1 to the SLC7A11 promoter (Fig. 6E).

Subsequently, we performed dual-luciferase reporter assays to evaluate the effect of YBX1 on SLC7A11 promoter activity. Compared to the luminescence intensity in cells co-transfected with the wild-type (wt) SLC7A11 promoter construct and empty vector (pcDNA3.1), YBX1 overexpression significantly increased relative luminescence intensity, demonstrating that YBX1 binds and activates the SLC7A11 promoter. When cells were co-transfected with a mutant SLC7A11 promoter reporter plasmid and YBX1-overexpressing plasmid, luminescence intensity decreased significantly yet remained detectable, suggesting partial dependence of this regulation on the mutated binding site (Fig. 6F).To further validate YBX1-mediated regulation of SLC7A11 protein expression, we performed WB in Re-AGS and Re-HGC27 cells with or without YBX1 knockdown (sh-YBX1). Under 5-Fu treatment, YBX1 protein bands were attenuated upon sh-YBX1 knockdown in both cell lines, confirming efficient knockdown. Concurrently, SLC7A11 protein band intensity markedly decreased, whereas GAPDH expression remained stable. These results demonstrate that YBX1 positively regulates SLC7A11 protein expression, and this regulatory effect persists under 5-Fu treatment (Fig. 6G). Collectively, integrating transcription factor prediction, bioinformatic analysis, and experimental validation, we conclusively establish that YBX1 promotes SLC7A11 protein expression by directly binding to its chromatin region and activating promoter activity.

Based on our initial hypothesis, we posited that ODC1 might play a significant role in regulating SLC7A11 transcription rather than through direct protein-level interactions. However, while we have preliminarily established that SLC7A11 transcription is enhanced via YBX1-mediated regulation, the precise function of ODC1 in SLC7A11 transcriptional regulation remains undefined. We therefore propose that YBX1 serves as a critical bridge facilitating ODC1’s influence on ferroptosis and drug resistance phenotypes through SLC7A11. Furthermore, we hypothesize that ODC1 may interact with YBX1 at the protein level. To identify ODC1-interacting proteins, we first performed Co-IP assays in Re-AGS and Re-HGC27 cells, followed by SDS-PAGE analysis.The Input lane represents total cellular protein, while immunoprecipitation (IP) using antibodies against either the exogenously expressed Flag-tagged protein fragment or endogenous ODC1 enriched potential protein complexes, establishing the foundation for subsequent identification. Intriguingly, to identify immunoprecipitated proteins, we employed mass spectrometry (MS) to measure the mass-to-charge ratios (m/z) of peptide fragments. The total ion chromatogram (TIC) in Fig. 6J displays retention times and intensities of various peptides, and the extracted ion chromatogram (EIC) of the peptide “AADPPAENSSAPEAEQGGAE” further confirmed the presence of YBX1—a critical finding for verifying interacting proteins.To validate the direct interaction between YBX1 and ODC1, we performed co-IP assays (Fig. 6I). When YBX1 was immunoprecipitated, Flag-tagged protein was detected by immunoblotting (IB). Conversely, immunoprecipitation with Flag antibody successfully pulled down YBX1, while IgG controls showed no nonspecific binding, confirming a specific interaction between YBX1 and the Flag-tagged target protein. Furthermore, we utilized the AlphaFold platform (https://alphafold.com/) to model potential three-dimensional structures of YBX1 and ODC1. The blue regions in the model represent high-confidence predictions with substantial reliability, providing structural support for potential binding modes between YBX1 and ODC1 molecules (Fig. 6H).

Subsequently, we analyzed the domain characteristics of ODC1 and YBX1 based on NCBI transcript information to establish a molecular foundation for studying their protein interaction mechanism. First, we identified four non-predicted transcripts of the ODC1 gene in NCBI and ultimately selected NM_002539.3 (full-length 1257 bp, encoding 427 aa). This gene contains only one predicted domain: PLPDE_III_ODC (37–408 aa). Its N-terminal flexible region (1–36 aa) may participate in protein localization. In contrast, YBX1 has a single non-predicted transcript (encoding 324 aa), with its core domain being the cold shock domain (CSD). According to domain architecture, we designed two truncated variants: CSD (1–128 aa) and CSD (129–324 aa).After constructing the truncated variants, we performed domain schematic analysis and co-IP assays. The results revealed detectable HA-tagged protein expression in IP complexes from both full-length YBX1 and YBX1 (1–128aa) groups, indicating ODC1-YBX1 binding in these two conditions. Similarly, HA signal was observed in co-IP assays with full-length ODC1 and ODC1 (37–408aa), confirming interaction occurrence in these groups. Thus, we identified the critical domain regions mediating the YBX1-ODC1 interaction: ODC1 utilizes its “PLPDE_III_ODC” domain (aa 37–408), while YBX1 depends on its cold shock domain (CSD, 1-128aa) for binding (Fig. 6K). Subsequently, immunofluorescence co-localization assays revealed significant subcellular co-distribution of YBX1 and ODC1 within cells, as evidenced by overlapping fluorescence signals in merged channels. This spatial co-localization provides additional support for their direct interaction (Fig. 6L).

### The YBX1–SLC7A11 axis governs ferroptosis

**Fig. 7:**
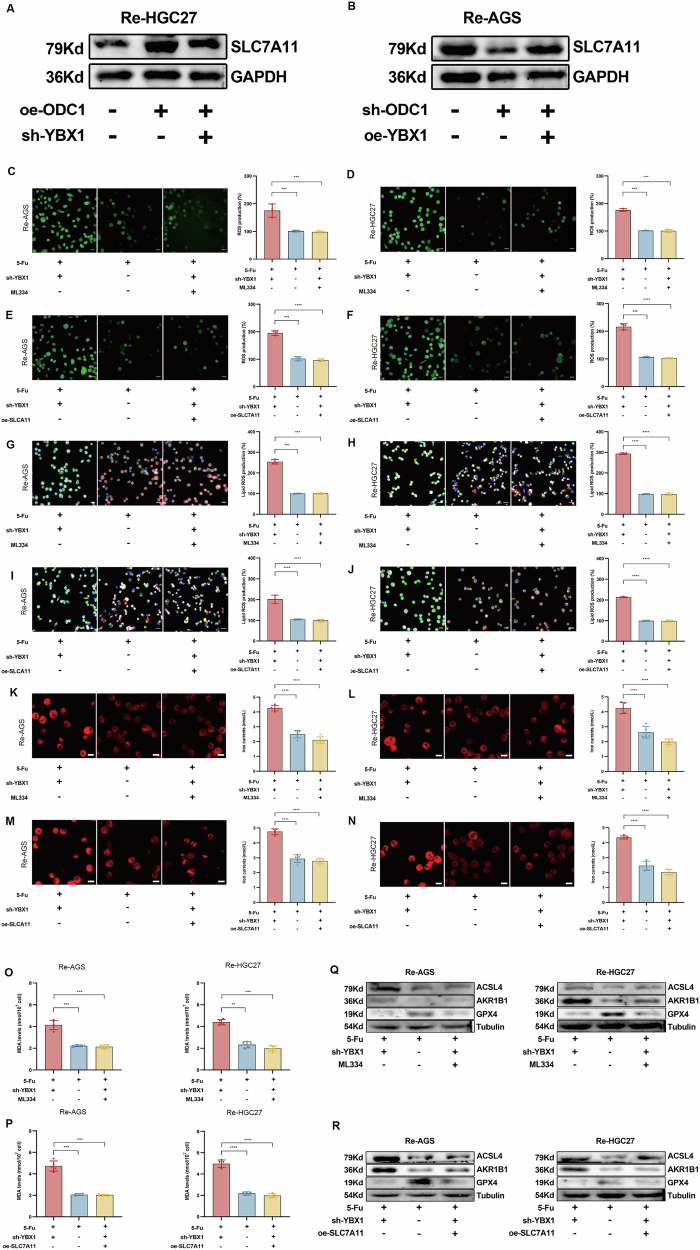
YBX1 modulates ferroptosis via SLC7A11 regulation. **A**, **B** Western blot demonstrates manipulating ODC1 and YBX1 expression (overexpression or knockdown) reciprocally regulates SLC7A11 protein levels in both Re-HGC27 and Re-AGS. **C**, **D** ML334 reverses sh-YBX1 ferroptosis by detection the level of ROS using DCF-DA staining.bars=20μm. **E**, **F** oe-SLC7A11 reverses sh-YBX1 ferroptosis by detection the level of ROS using DCF-DA staining.bars = 20 μm. **G, H** ML334 reverses sh-YBX1 ferroptosis by detection the level of Lipid ROS using C11-BODIPY581/591 staining.bars=20 μm. **I, J** oe-SLC7A11 reverses sh-YBX1 ferroptosis by detection the level of Lipid ROS using C11-BODIPY581/591 staining.bars=20 μm. **K, L** ML334 reverses sh-YBX1 ferroptosis by RhoNox-6 fluorescence and Ferrous iron assay kit.bars = 20 μm. **M, N** oe-SLC7A11 reverses sh-YBX1 ferroptosis by RhoNox-6 fluorescence and Ferrous iron assay kit.bars = 20 μm. **O, P** ML334 and oe-SLC7A11 reverses sh-YBX1 ferroptosis by MDA assay kit. **Q, R** ML334 and oe-SLC7A11 reverses sh-YBX1 ferroptosis by as determined by analyzing ferroptosis-related protein expression via Western blot.*P < 0.05, **P < 0.01, *** P < 0.001,****P < 0.0001.

Finally, we designed WB experiments to validate the synergistic regulation of SLC7A11 protein expression by ODC1 and YBX1 in Re-HGC27 and Re-AGS cells. In Re-HGC27 cells, ODC1 overexpression (oe-ODC1) upregulated SLC7A11, whereas concurrent knockdown of YBX1 (sh-YBX1) attenuated this upregulation. In Re-AGS cells, ODC1 knockdown (sh-ODC1) downregulated SLC7A11, while simultaneous YBX1 overexpression (oe-YBX1) partially rescued SLC7A11 expression. These results demonstrate that YBX1 and ODC1 directly interact through specific structural domains and cooperatively regulate the expression of downstream target protein SLC7A11 (Fig. 7A, B).

Based on the aforementioned findings, we propose that YBX1 regulates SLC7A11 expression. However, whether YBX1 participates in the ferroptosis pathway and thereby indirectly influences STAD drug resistance remains unclear. Moreover, we sought more reliable evidence to directly confirm that YBX1 modulates ferroptosis through SLC7A11. Therefore, we refocused our investigation on ferroptosis-related phenotypes. To establish a definitive correlation between YBX1 and ferroptosis, we introduced the small-molecule compound ML334 in our experiments. ML334 is a potent and selective GPX4 agonist that enhances GPX4 enzymatic activity via allosteric activation. This implies that ML334 binds to a non-active site of GPX4, altering its protein conformation to facilitate catalytic reactions. Consequently, it augments cellular capacity to eliminate lipid peroxides, thereby suppressing or inhibiting ferroptosis [41, 42]. To ensure accurate interpretation of experimental results, we performed replicate experiments in both drug-resistant cell lines. Upon treatment with 5-Fu alone, the drug-resistant cells exhibited relatively low ROS accumulation due to their inherent drug resistance. Following this baseline assessment, YBX1 knockdown significantly increased cellular ROS accumulation. However, this effect was markedly reversed upon co-administration of ML334, which concurrently attenuated stimulus-induced cell death.These data strongly demonstrate that YBX1 knockdown-induced cell death is ferroptosis-dependent (Fig. 7C, D). Furthermore, to further validate the conclusion that YBX1 exerts its biological activity through regulating SLC7A11, we conducted parallel experiments under 5-Fu stimulation. We observed that the YBX1 knockdown-induced increase in cellular ROS accumulation was reversed by SLC7A11 overexpression. This indicates that SLC7A11 is the critical molecular mediator between YBX1 and ferroptosis, thereby confirming our previous hypothesis that YBX1 modulates ferroptosis via SLC7A11 (Fig. 7E, F). Subsequently, we performed BODIPY assays to quantify lipid reactive oxygen species (LIPROS) levels. Results aligned with ROS data: fluorescence microscopy revealed enhanced green fluorescence intensity (oxidized probe) at 510 nm after YBX1 knockdown, indicating elevated lipid peroxidation. Both cell lines exhibited significant LIPROS accumulation.Following ML334 treatment, green fluorescence intensity was reversed while red fluorescence intensity substantially increased (Fig. 7G, H). This reversal phenotype was also reproduced upon SLC7A11 overexpression (Fig. 7I, J). Next step,we assessed ferrous iron and MDA, two critical mediators in ferroptosis. Given that ML334 bypasses the GSH system by directly targeting its downstream effector GPX4, GSH levels are no longer a determinant of cell fate in this context. Consequently, we did not measure GSH concentrations in this experimental phase. Both ferrous iron quantification (using iron assay kits or RhoNox-6 staining) and MDA measurements yielded results consistent with the aforementioned lipid peroxide data (Fig. 7K–P). At the protein level, WB analysis revealed that both ACSL4 and AKR1B1—positively correlated with ferroptosis—exhibited reversal of their YBX1-knockdown-induced upregulation following ML334 intervention or SLC7A11 overexpression. Conversely, GPX4 displayed an opposite pattern (Fig. 7Q, R). Collectively, our findings demonstrate that YBX1 knockdown, when counteracted by either ML334 or SLC7A11 overexpression, effectively suppresses 5-Fu-induced elevation of LIPROS and other oxidative markers in Re-AGS and Re-HGC27 cells. These data establish that YBX1 regulates ferroptosis levels through SLC7A11.

### Erastin Promotes the Sensitivity of YBX1-Overexpressed and Chemoresistant STAD to 5-Fu Treatment

**Fig. 8:**
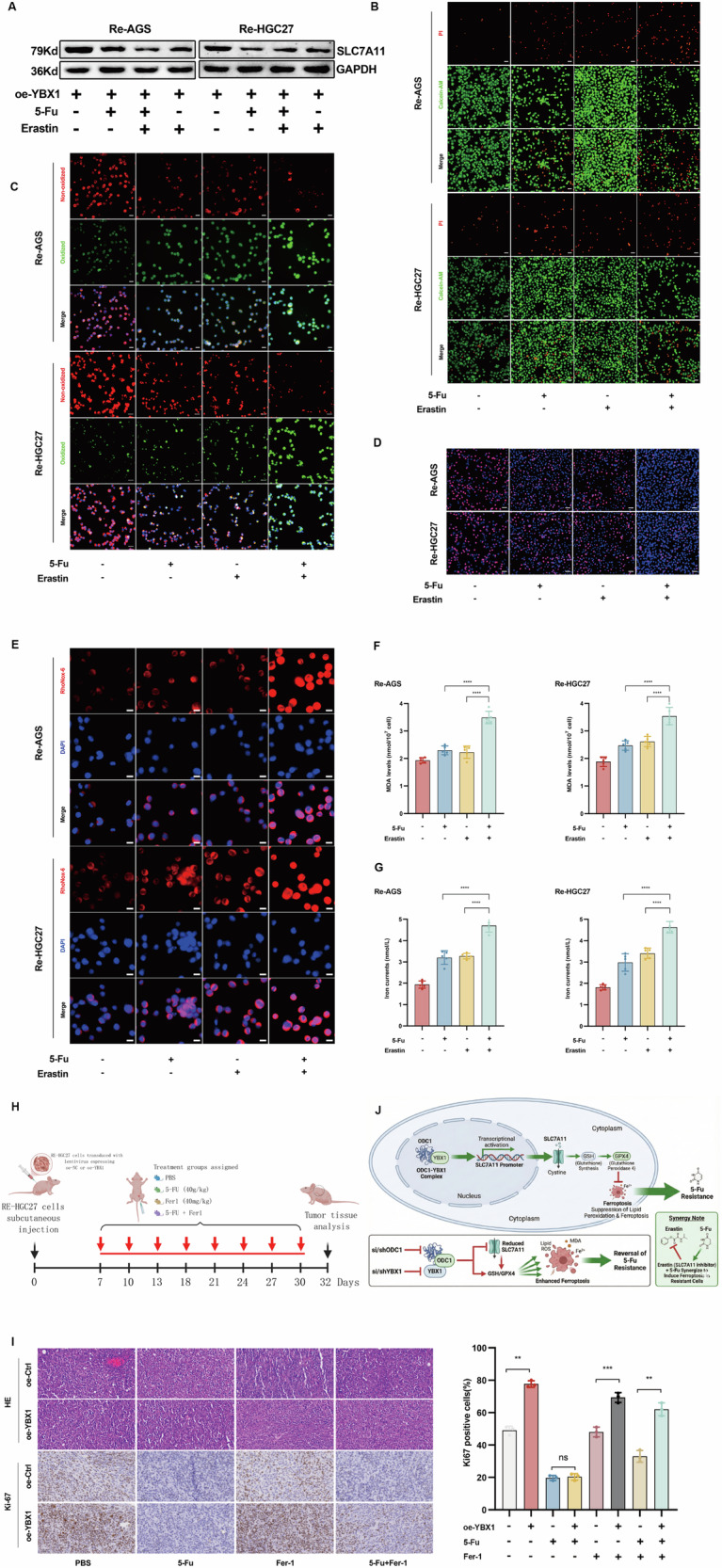
SLC7A11 inhibition overcomes YBX1-mediated chemoresistance in vitro and in vivo. **A** Western blot analysis for SLC7A11 protein expression levels in cells treated as shown. **B** Calcein-AM/PI staining was performed to test cell death treated as shown. **C** The level of Lipid ROS detected by C11-BODIPY581/591 staining in the condition as shown. **D** Cell proliferation in each treatment group was detected by the EDU assay. **E**, **G** Iron dysregulation detected by RhoNox-6 fluorescence and Ferrous iron assay kit in the condition as shown.scale bars=20μm. **F** MDA levels by MDA assay kit in the condition as shown. **H**, **I** Schematic diagram of the in vivo experimental workflow, along with H&E staining and Ki67 immunohistochemistry performed on tumors harvested from the different treatment groups. **J** The graphical abstract illustrates the proposed mechanism by which ODC1 regulates ferroptosis.*P < 0.05, **P < 0.01, *** P < 0.001, ****P < 0.0001.

To further investigate whether ferroptosis is involved in the drug sensitivity of gastric adenocarcinoma cells to 5-Fu, we treated Re-AGS and Re-HGC27 cells with Erastin, a SLC7A11 inhibitor. WB analysis revealed that compared with the untreated control group,the YBX1-overexpression-induced upregulation of SLC7A11 was reversed by either Erastin or 5-Fu monotherapy. Significantly, combinatory treatment with Erastin and 5-Fu resulted in substantially reduced SLC7A11 protein expression levels (Fig. 8A). Furthermore, we discovered that Erastin potently enhanced 5-Fu-stimulated lipid ROS production in chemo-resistant cells with high YBX1 expression (Fig. 8C). Concurrently, using comprehensive assay kits, we detected that co-incubation with Erastin significantly elevated 5-Fu-triggered MDA levels and intracellular iron content (Fig. 8E-G). Subsequently, we refocused our investigation on chemoresistance. EDU assays demonstrated that despite inherent drug resistance, Erastin treatment significantly suppressed cellular proliferation. Moreover, escalating 5-Fu concentrations markedly reduced cell viability following Erastin exposure (Fig. 8B, D). Based on these in vitro models and experimental evidence, we demonstrate that Erastin remains capable of inducing ferroptosis in chemo-resistant gastric cancer cells—even with coexisting YBX1 overexpression —thereby enhancing 5-Fu sensitivity and ultimately triggering cell death.To further validate our aforementioned conclusions, we established animal models for in vivo experimentation. Nude mice were inoculated with Re-HGC27 cells transduced for YBX1 overexpression or control vectors. One week post-inoculation, established tumors were observed, followed by tri-weekly drug administration cycles. After eight cycles, tumors were harvested for hematoxylin and eosin (H&E) staining and Ki67 proliferation analysis(Fig. 8H). Notably, even in vivo, combined Erastin and 5-Fu treatment resulted in markedly decreased Ki67 staining intensity, confirming attenuated proliferative capacity. This demonstrates Erastin’s ability to enhance 5-Fu sensitivity in chemo-resistant cells within in vivo models (Fig. 8I).

Collectively, this study proposes and preliminarily validates a novel regulatory axis governing chemoresistance through ferroptosis modulation in gastric adenocarcinoma cells—namely, the ODC1-YBX1-SLC7A11-Ferroptosis-Chemoresistance axis (Fig. 8J)—based on integrated in vitro and in vivo evidence. This discovery may provide innovative therapeutic perspectives and strategies for gastric *adenocarcinoma* treatment, particularly for patients exhibiting pervasive chemoresistance.

## Discussion

Gastric adenocarcinoma remains a prevalent and challenging malignancy of the digestive system. The limited understanding of its pathogenesis and chemoresistance mechanisms continues to contribute to unsatisfactory clinical outcomes globally [43–45]. Identifying novel prognostic biomarkers and therapeutic targets is therefore essential for improving the management of advanced gastric cancer, particularly in chemoresistant patients. Metabolic reprogramming under hypoxia represents a hallmark of gastric cancer, profoundly influencing cancer cell proliferation, programmed cell death, nutrient trafficking, and therapy resistance [46]. As a pivotal enzyme in the ornithine cycle with close links to cellular metabolism, ODC1 has been implicated in the pathogenesis of various malignancies [21, 47, 48]. In this study, we observed significantly elevated ODC1 expression in gastric adenocarcinoma tissues compared with normal controls, consistent with previous reports [49]. Kaplan–Meier survival analysis further revealed that high ODC1 expression predicted poorer disease-free survival (DFS) and overall survival (OS). Genetic ablation of ODC1 markedly attenuated the proliferative, migratory, and invasive capacities of gastric adenocarcinoma cells in vitro, a finding corroborated in animal models. Transcriptomic sequencing unexpectedly indicated that ODC1’s functional scope extends beyond polyamine metabolism to include drug response regulation, suggesting its potential role in chemoresistance. Using two established 5-Fu-resistant gastric adenocarcinoma cell lines, we confirmed ODC1 upregulation under resistance conditions. Strikingly, ODC1 knockdown reversed the chemoresistant phenotype, supporting its functional involvement in drug resistance through mechanisms that were previously unclear.Notably, when cell death was triggered through targeted inhibition, ferroptosis emerged as the predominant form of death among all modalities examined. Ferroptosis is an iron-dependent regulated cell death driven by uncontrolled lipid peroxidation on plasma and organellar membranes [50]. We demonstrated that ODC1 knockdown via shRNA robustly induced intracellular ROS accumulation, lipid peroxidation, elevated MDA content, increased iron levels, and reduced glutathione (GSH)—even in the absence of 5-Fu treatment in chemoresistant cells. Subsequent mass spectrometry and co-immunoprecipitation assays revealed that ODC1 physically interacts with YBX1, a multifunctional DNA/RNA-binding protein, to transcriptionally regulate the expression of SLC7A11, a key subunit of system Xc− that imports cystine for glutathione synthesis and redox maintenance [34]. We provide multifaceted evidence that ODC1, together with YBX1, maintains intracellular redox homeostasis and supports tumor proliferation by activating SLC7A11 transcription. YBX1 was further established as a central regulator of ferroptosis via SLC7A11, as ML334—a SLC7A11 activator—effectively rescued sh-YBX1–induced ferroptosis suppression. Conversely, the ferroptosis inducer Erastin enhanced 5-Fu chemosensitivity in YBX1-overexpressing chemoresistant cells. Collectively, this study identifies and validates a previously unrecognized chemoresistance axis—the ODC1–YBX1–SLC7A11 signaling cascade—that promotes chemoresistance by repressing ferroptosis. These findings reveal exploitable therapeutic targets and propose actionable strategies for gastric cancer treatment, particularly in therapy-refractory populations.

ODC1 functions as the primary rate-limiting enzyme in mammalian polyamine biosynthesis, catalyzing the decarboxylation of ornithine to produce putrescine and thereby governing intracellular polyamine homeostasis. Sustained hyperactivation of ODC1 leads to dysregulated polyamine accumulation, a recognized hallmark of oncogenic transformation [51]. Polyamines—ubiquitously expressed organic cations in nearly all eukaryotes—exert multifaceted biological functions, including the regulation of gene expression, cellular proliferation, apoptosis, and cell cycle progression [52]. Seminal work by Cleveland et al. identified ODC1 as the first transcriptional target of c-MYC, positioning it as a versatile effector in malignant pathogenesis due to its broad expression in proliferative tissues [53]. Chemotherapy has also been shown to induce breast cancer stem cell enrichment through HIF-1α–mediated upregulation of polyamine biosynthesis, wherein cytotoxic stress activates HIF-1 to elevate ODC1 expression [54]. Furthermore, in hepatocellular carcinoma, ODC1 remodels the acidic tumor microenvironment via the AKT/GSK3β/β-catenin signaling cascade, thereby promoting proliferation, migration, and invasion [55]. Consistent with these findings, our experiments validate that ODC1 upregulation critically drives gastric cancer progression. Suppression of ODC1 expression markedly attenuated malignant phenotypes, specifically the invasion and proliferative capacities of gastric adenocarcinoma cells. This tumor-promoting role was further corroborated in vivo using BALB/c nude mice implanted with ODC1-knockout gastric adenocarcinoma cells, which exhibited significantly inhibited xenograft tumor growth.Beyond gastric cancer, emerging mechanistic insights from small cell lung carcinoma (SCLC) reveal that arginine and its downstream polyamine metabolites hypersensitize cancer cells to ferroptosis in an H₂O₂-dependent manner. Specifically, iron overload activates ODC1 expression via the WNT/MYC signaling pathway, accelerating polyamine biosynthesis and establishing a self-amplifying feedforward circuit: ferroptosis–iron overload–WNT/MYC/ODC1–polyamine–H₂O₂, which collectively potentiates ferroptotic cell death [21]. Accordingly, we confirmed that shRNA-mediated ODC1 knockdown robustly induces an oxidative stress cascade characterized by intracellular ROS surge, aggravated lipid peroxidation, MDA accumulation, iron overload, and GSH depletion.

Ferroptosis represents a form of programmed cell death that is distinct from apoptosis and necrosis, primarily defined by iron-dependent lipid peroxidation that leads to the collapse of cellular antioxidant defenses and results in oxidative cell death [56]. Mechanistically, in iron-rich microenvironments, excess hydrogen peroxide reacts with ferrous iron via the Fenton reaction, producing highly reactive hydroxyl radicals. These radicals target bis-allylic sites in polyunsaturated fatty acid (PUFA)-containing phospholipids within cellular membranes, initiating lipid peroxidation that culminates in membrane disruption and ferroptotic death [57]. As an evolutionary adaptation, cells have developed robust defense mechanisms against ferroptosis, largely centered on the SLC7A11/GPX4-axis and the associated glutathione-based redox system [58]. In this study, second-generation sequencing data indicated a marked downregulation of SLC7A11 expression following ODC1 suppression, prompting the hypothesis of a functional interplay between these factors. However, subsequent co-immunoprecipitation assays yielded negative results, ruling out direct protein-protein interaction and shifting the focus to the transcriptional regulation of SLC7A11 by ODC1. Functionally, SLC7A11 forms a highly specific transmembrane channel through 12 membrane-spanning helices, facilitating the uptake of extracellular cystine. This cystine is subsequently reduced to cysteine in an NADPH-dependent manner, providing a rate-limiting substrate for glutathione synthesis and thereby conferring indirect protection against ferroptosis [29]. Previous studies have linked SLC7A11 to gliomagenesis and nasopharyngeal carcinoma development [59, 60], and emerging evidence indicates that SLC7A11 activation may expose new therapeutic vulnerabilities in gastric adenocarcinoma—a perspective aligned with the present findings [45]. The expression and activity of SLC7A11 are tightly regulated through multiple layers of control, with transcriptional and epigenetic mechanisms playing predominant roles in maintaining redox homeostasis [61, 62]. By integrating JASPAR-based transcription factor prediction, CUT&Tag genomic profiling, and dual-luciferase reporter assays, we identified YBX1 as a bona fide transcriptional regulator of SLC7A11 that enhances ferroptosis resistance in drug-adapted cancer cells. YBX1, a multifunctional DNA/RNA-binding protein member of the evolutionarily conserved cold-shock domain superfamily, coordinates a range of nucleic acid-related processes including transcription, DNA repair, mRNA stability, splicing, and translation [36, 63]. At the cellular level, it regulates key processes such as proliferation, differentiation, autophagy, stress response, and tumorigenesis [37, 38]. Notably, the involvement of YBX1 in modulating ferroptosis to affect 5-fluorouracil resistance in gastric adenocarcinoma had not been previously established. Here, we demonstrate that YBX1 promotes chemoresistance by regulating ferroptotic pathways, and rescue assays definitively identified SLC7A11 as the mechanistic effector responsible for this regulatory axis.

It is well established that current chemotherapeutic regimens for gastric cancer primarily include platinum derivatives, fluoropyrimidines, and taxanes. 5-Fluorouracil (5-Fu) has been shown to trigger intracellular ROS generation, leading to programmed cell death under conditions of oxidative stress [64]. This mechanism shares fundamental characteristics with ferroptotic pathways, revealing an intrinsic link between chemoresistance and susceptibility to ferroptosis. Notably, both prolonged exposure and supra-physiological levels of ROS can enhance chemoresistance in gastric cancer cells [65]. Paradoxically, although agents such as oxaliplatin and irinotecan induce substantial ROS accumulation, tumor cells activate compensatory glutaminolysis to counteract oxidative damage—a key mechanism underlying chemoresistance [66]. Consequently, inducing ferroptosis has emerged as a promising strategy to overcome chemoresistance. Studies have shown that simultaneous inhibition of STAT3 and activation of ATF3 disrupts pathways that suppress ferroptosis, thereby inhibiting gastric tumor growth and reducing chemotherapy resistance [67]. From a metabolic perspective, targeted inhibition of lipoxygenases (ALOXs) prevents the accumulation of lipid peroxides, promoting ferroptotic cell death in gastric adenocarcinoma [68]. In this study, we demonstrate that ODC1 regulates SLC7A11 expression through the transcription factor YBX1, thereby modulating chemoresistance via ferroptosis pathways in gastric adenocarcinoma cells. Therefore, therapeutic targeting of ODC1 to suppress SLC7A11 and enhance ferroptosis represents a rational strategy for improving the efficacy of 5-Fu-based chemotherapy.

## Conclusion

Current therapeutic outcomes for gastric adenocarcinoma remain suboptimal, a challenge largely attributable to tumor immune evasion and the establishment of chemoresistance mechanisms Importantly, we preliminarily demonstrate that ODC1 overexpression correlates with chemotherapy resistance in gastric adenocarcinoma patients. ODC1 inhibition consistently sensitizes gastric adenocarcinoma cells to both ferroptosis inducers and chemotherapeutic agents in vitro and in vivo, providing mechanistic validation for ODC1-targeted therapies while directly confirming the existence of the ODC1-YBX1-SLC7A11-ferroptosis-chemoresistance axis. Future drug discovery efforts should prioritize developing ODC1-specific inhibitors—a strategy poised to expand the therapeutic arsenal against gastric and other malignancies.

## Materials and Methods

### Human Tissue Samples and Tissue Microarrays

Anonymized human gastric tissue specimens (April 2018–October 2020) were obtained from Northern Jiangsu People’s Hospital Affiliated to Medical School of Nanjing University with approval from its Institutional Ethics Committee. All participants provided written informed consent compliant with the Declaration of Helsinki and its subsequent amendments.Complete clinical information-including general baseline data and pathological staging-was available for all patients, with each undergoing a comprehensive 3-year follow-up.We constructed five tissue microarrays (TMAs) comprising 242 paired tumors and matched adjacent normal tissues.

### Cell lines and cell culture

The human gastric epithelial cell line GES-1 and gastric cancer cell lines NCI-N87, AGS, SUN-719, MKN-1, MKN-45, SUN-484, and HGC-27 were sourced from ProCell Life Science & Technology Co., Ltd. (Wuhan, China). NCI-N87, SUN-719, SUN-484, HGC-27, MKN-1, and MKN-45 were maintained in RPMI-1640 supplemented with 10% fetal bovine serum (FBS) and 1% penicillin-streptomycin (100 U/mL penicillin, 100 μg/mL streptomycin); AGS cells were cultured in Ham’s F-12 medium with 10% FBS and 1% penicillin-streptomycin; GES-1 was grown in DMEM containing 10% FBS and 1% penicillin-streptomycin. Chemoresistant sublines Re-AGS and Re-HGC27 were established from their parental counterparts: Re-AGS propagated in Ham’s F-12 containing 20 μM 5-Fu, and Re-HGC27 sustained in RPMI-1640 with 15 μM 5-Fu. All cell lines were incubated at 37 °C under 5% CO_2_in a humidified atmosphere, with monthly mycoplasma contamination screening.

### Methodology for Culturing Drug-Resistant Cell Lines

Resistant subline establishment involved initial 24-hour exposure of parental cells to 1 μM 5-Fu in RPMI-1640 or Ham’s F-12 medium. Residual drug was eliminated through triplicate phosphate-buffered saline (PBS) washes before reversion to chemotherapeutic-free complete medium. Following daily viability monitoring until <5% mortality with reestablished proliferation kinetics, pulsed treatments were cyclically administered at 2-3 week intervals contingent upon confluency. Progressive selection pressure was applied through dose-escalation (1.5-2-fold increments post-adaptation). Post 6-month induction including a terminal 2-week drug-free period, stable resistant variants (designated Re-AGS/Re-HGC-27) were validated by resistance indices (RI = IC₅₀ resistant/IC₅₀ parental) exceeding 2.0.

### Reverse Transcription Quantitative PCR (RT-qPCR) Analysis

Total RNA was isolated with TRIzol® (Thermo Fisher Scientific) per standard protocols. Spectrophotometric quantification verified RNA integrity (A260/A280 > 1.8). Complementary DNA (cDNA) synthesis employed RevertAid First Strand cDNA Synthesis Kit (Thermo Fisher Scientific) with oligo(dT) primers. Gene-specific primers were designed using PrimerBank (https://pga.mgh.harvard.edu/primerbank/). Amplification reactions utilized SYBR Green I qPCR Kit chemistry (Aibotek, Wuhan, China; Cat# RK21203) on a StepOnePlus™ thermocycler (Applied Biosystems, Thermo Fisher Scientific). Relative quantification normalized to GAPDH was computed via the 2^−ΔΔCq approach. Technical triplicates ensured reproducibility.The primer sequences used are provided in Supplementary Table 2.

### Western Blot and Silver Staining

Protein quantification was performed with BCA protein assay kit (Vazyme, Nanjing, China). After loading equivalent protein quantities, electrophoretic separation employed SDS-PAGE gels followed by wet transfer to PVDF membranes (Merck, Singapore). Membranes underwent 1.5 hour blocking in 5% bovine serum albumin (BSA; Solarbio, Beijing, China) at ambient temperature prior to overnight 4 °C exposure to primary antibodies. Subsequent to triple TBST rinses (10 min/wash), species-matched HRP-conjugated secondary antibodies were applied at room temperature for 2 h. Chemiluminescent detection was performed using an Enhanced chemiluminescence (ECL) (Biosharp, Hefei, China) with signal captured on an Azure c600 imaging system.For silver staining, post-electrophoretic protein separation, Rapid Silver Staining Kit (Beyotime, P0017S) procedures were executed: gel fixation (30% ethanol/10% acetic acid), ultrapure water washes, 0.02% sodium thiosulfate sensitization, and 0.2% silver nitrate staining. Development utilized kit-supplied reagents per manufacturer specifications. All aqueous solutions originated from Milli-Q purification systems.The antibodies used are provided in Supplementary Table 3.

### Immunohistochemistry Staining

Formalin-fixed paraffin-embedded (FFPE) sections underwent dewaxing in Histo-Clear® (National Diagnostics, HS-202), followed by hydration through graded ethanols. Antigen retrieval utilized 10 mM citrate buffer (pH 6.0) with microwave heating. Endogenous peroxidases were inhibited by 3% H₂O₂ treatment prior to 1-h blocking in 5% BSA. Primary antibodies were applied overnight at 4 °C. After triple PBS washes, HRP-conjugated streptavidin detection (ZSGB-BIO, PV-9000) proceeded for 20 min at ambient temperature. Nuclear counterstaining employed Mayer’s hematoxylin (MaiXin Biotechnology).Digital whole-slide images were acquired using an Olympus FSX100 microscope (Tokyo, Japan) with UC90 20×/0.75 NA objective. Two independent pathologists blinded to clinical data performed immunohistochemical scoring per established methodology. For each section, five random fields were assessed using a dual-parameter system: (1) Staining intensity: 0 (None), 1+ (Weak/pale yellow), 2+ (Moderate/yellow-brown), 3+ (Strong/brown).(2)Positive cell percentage: 0 ( ≤ 5%), 1+ (6–25%), 2+ (26–50%), 3+ (≥51%).Total scores were calculated as (Intensity score) × (Percentage score) (range 0–9), with specimens scoring ≥1 classified as positive expression cases.The antibodies used are provided in Supplementary Table 3.

### TEM

Mitochondrial ultrastructure was analyzed by transmission electron microscopy (TEM). Following exposure to 20 μM 5-Fu, the stably resistant Re-AGS and Re-HGC27 cell lines were fixed in 2.5% glutaraldehyde (in PBS, pH 7.4) at 4 °C for 2.5 h. After washing with PBS, the samples were post-fixed in 1% osmium tetroxide at 4 °C for 2 h. Subsequently, the samples were dehydrated in a graded ethanol series, embedded in Spurr’s resin, and sectioned. The ultrathin sections were stained with uranyl acetate and lead citrate prior to TEM observation.

### Cell transfection

Lentiviral constructs encoding genes-targeting shRNAs and matched control vectors were procured from GeneChem Co., Ltd. (Shanghai, China). AGS/Re-AGS and HGC27/Re-HGC27 gastric cancer cells were transduced with respective lentiviruses (sh-genes or negative control). The sh-genes targeting sequence is documented in Supplementary Table 2. Transduced populations underwent puromycin selection (2 μg/mL) over 2 weeks to establish stable knockdown lineages. Silencing efficiency was assessed through Western blot analysis.

### Immunofluorescence Staining

Fixed in 4% paraformaldehyde, cell specimens underwent permeabilization using 0.5% Triton X-100 followed by 5% BSA blocking to mitigate nonspecific binding. Primary antibodies were incubated overnight at 4°C. After PBS washing (3×), samples in confocal dishes exposed to Alexa Fluor 488/555/594-conjugated secondary antibodies (1:200 dilution; Beyotime, A0453, A0423; Abcam, AB175473) for 1-hour dark incubation with nuclear counterstaining using DAPI (Solarbio life sciences, C0060; Beijing, China). Sequential confocal imaging was performed on a Zeiss LSM 800 microscope (Germany) via 63× oil immersion objective to preclude spectral overlap.

### Cell proliferation assay

Cell proliferation was quantified using the Cell Counting Kit-8 (CCK-8; Yeasen, Shanghai) per manufacturer protocols. Sh-Ctrl and shRNA-transfected cells were plated in 96-well plates (Corning, USA) at 1 × 10³ cells/well. At designated intervals (24, 48, 72, 96,120 h), 10 μL CCK-8 solution was added to each well and incubated for 2 h at 37 °C/5% CO₂ atmosphere. Absorbance at 450 nm was subsequently measured via BioTek Synergy microplate reader (Winooski, VT, USA) to determine proliferation rates.

For the colony formation assay,cells (5 × 10²/well) from each group were plated in 6-well plates (Corning, USA) and cultured for 14 days at 37 °C/5% CO₂. Colonies were subsequently fixed with 4% paraformaldehyde (10 min), stained with 5% crystal violet (Solarbio, Beijing), and air-dried. Colonies containing ≥50 cells were quantified across three biological replicates. Data represent mean ± standard deviation (SD) of triplicate determinations.

Chamber slide-seeded cells were pulsed with 10 μM EdU (2 h), fixed/permeabilized, and detected using Alexa Fluor 594-based EdU Cell Proliferation Kit (Beyotime C0078L), with DAPI nuclear counterstaining. Images were captured on a Zeiss LSM 900 confocal microscope (40× objective), and EdU+ cell percentages were quantified from ≥5 fields via ImageJ.

### Wound healing and Transwell assays

Wound healing:Gastric cancer cells in 6-well plates reached ~85% confluency before generating linear scratches with sterile 200 μL pipette tips. After washing with PBS to remove debris, phase-contrast microscopy (Olympus IX83) documented wound closure at 0/24/48 h. Closure percentage (relative to initial gaps) was determined from triplicate measurements at randomized sites per wound.

Transwell Migration and Invasion Assays:Cells transfected (5 × 10⁴) in serum-free medium were seeded into Transwell upper chambers (8 μm pores; Corning #3422) within 24-well plates. Invasion assays used inserts pre-coated with diluted Matrigel™ (Vazyme GL201; 1:8 serum-free dilution), while migration assays employed uncoated membranes. Lower chambers contained 25% FBS chemoattractant. After incubation (migration: 24 h, invasion: 48 h), non-migratory cells were removed. Migrated/invaded cells on membrane undersides were fixed with 4% paraformaldehyde, stained in 0.5% crystal violet, and quantified in ≥3 random fields/insert (200×, Olympus IX83). Triplicates were performed per condition.

### Cell cycle assay

Cell cycle distribution was assessed by flow cytometry. Cells were harvested, washed twice with phosphate-buffered saline (PBS), digested with trypsin, and fixed in 70% ethanol for 4 h at 24°C. Fixed cells were then stained with 500 μL propidium iodide (PI) solution (Yeasen, Shanghai, China) for 30 min at 37 °C. DNA content quantification was performed using a BD LSRFortessa flow cytometer (BD Biosciences, USA), with phase distribution analysis (G1, S, G2/M) conducted in FlowJo software (v10.9).

### Co-immunoprecipitation and Mass Spectrometry Analysis

Conducting co-immunoprecipitation (Co-IP) screened for putative protein interaction partners.

Cells were lysed in IP buffer (30 min, ice) and centrifuged (14,000 × g, 15 min, 4 °C). Pre-cleared supernatants underwent overnight incubation with 5–10 ug primary antibody or control IgG at 4 °C, followed by 2-h binding with protein A/G magnetic beads (Vazyme PB201) under rotation. After rigorous washing, protein complexes were either boiled in SDS loading buffer for elution or processed via on-bead digestion. Samples requiring mass spectrometry were submitted to BGI Group (Shenzhen, China) for LC-MS/MS analysis.

### Dual-Luciferase Reporter Assay

293 T cells (1 × 10⁵ cells/well) plated in 48-well dishes underwent LipoCR (Beyotime) transfection at 70–80% confluency. Co-transfection included either ΔPro, Pro-WT, or binding site mutant (mut1/mut2) firefly luciferase reporter constructs paired with control or RELA expression vectors. Post 72 h incubation, luciferase activity was quantified per manufacturer’s protocol (Dual-Luciferase Reporter Assay Kit, Beyotime RG088S) using firefly/Renilla luminescence ratios. Triplicate datasets were acquired.

### CUT&Tag Assay

The High-Sensitivity CUT&Tag Assay Kit (Vazyme TD904) was employed per manufacturer protocol. AGS cells (1 × 10⁶) underwent two washes with 1.5 mL Wash Buffer, followed by TF Enhancer-mediated crosslinking to stabilize transcription factor-DNA complexes. Subsequent concanavalin A (ConA) magnetic bead binding proceeded for 15 min at room temperature(RT). Sequential incubations included: primary antibody (overnight at 4 °C) and matched secondary antibody (1 h, RT). pAG-Tn5 transposase facilitated targeted tagmentation during 60-min incubation. Tagmentation activated with specialized buffer (1 h) ceased using stop buffer (10 min, 55 °C). Crosslink reversal occurred at 80 °C for 2 h. Extract Beads Pro purified DNA fragments underwent 18-cycle PCR amplification prior to Illumina NovaSeq sequencing (Nanjing Jiangbei Center).

### Calcein-AM and propidium iodide (PI) assays

Cells viability was evaluated per manufacturer protocol using a Calcein-AM/PI Double Staining Kit (Yeasen, 40747ES76, China). Treated test-condition were incubated with a solution containing 2 mM Calcein-AM (5 μl) and 1.5 mM PI (15 μl) diluted in 5 mL assay buffer. After 30-min incubation and PBS washing, Zeiss LSM 800 microscope (Germany) software performed imaging and quantification.

### ROS quantification

Intracellular ROS levels were measured using the fluorescent probe H2DCFDA (2’,7’-dichlorodihydrofluorescein diacetate; Proteintect, CM01378,Wuhan,China). A 10 mM stock solution (in DMSO) was prepared and diluted prior to experiments. Adherent cells underwent 30-min dark incubation at 37 °C with 5 μM H2DCFDA in PBS.Zeiss LSM 800 microscope (Germany) software performed imaging and quantification.

### Monitoring Changes in Intracellular Lipid Peroxidation

Prepare BODIPY 581/591 C11(Beyotime,S0043S) working solution (2 μM) by diluting an aliquot of 2 mM BODIPY 581/591 C11 in cold PBS at 1:1000 dilution (1 µl stock per 1 ml PBS). Add 1 ml working solution per well of six-well plates. Incubate cells at 37 °C for 10-30 min. Wash twice with PBS, then stain with DAPI in dark for 10 min before imaging and quantification by Zeiss LSM 800 microscope (Germany) .

### Detection of Intracellular Ferrous Ions

Prepare RhoNox-6 staining solution(Beyotime,S1070S) by combining 0.1 µL RhoNox-6 (1000X), 0.1 µL Hoechst 33342 (1000X), and 100 µL serum-free medium per reaction. After washing cells three times with PBS, add 1 mL of the staining solution per well to six-well plates. Incubate at 37 °C under light-protected conditions for 30 minutes before imaging and quantification by Zeiss LSM 800 microscope (Germany) .

Ferrous ion levels quantified using an Iron Assay Kit (Solarbio, BC5415,Beijing) according to manufacturer protocols. Cells were lysed to release intracellular Fe^2+^. After centrifuging at 12,000 g/10 min, the supernatant underwent heating (95°C, 5 min) in working buffer followed by rapid-cooling in ice water. Chloroform (60 µL) was added to 200 µL of supernatant, followed by vortexing and centrifugation. The upper aqueous phase was immediately analyzed for absorbance at 520 nm using a microplate reader.

### Ferroptosis-Related Markers Profiling (Assays)

MDA detection:Transfer 5 million cells into a centrifuge tube and discard the supernatant after centrifugation. Add 1 mL extraction buffer, then disrupt bacterial/cellular structures via sonication (parameters: 200 W power, 3-sec bursts, 10-sec intervals, 30 cycles). Centrifuge at 8,000 * g for 10 min at 4 °C. Carefully collect the supernatant and place on ice for subsequent assays. Prepare the working solution according to malondialdehyde (MDA) assay instructions (Solarbio, BC0025,Beijing), mix with samples, and incubate in boiling water for 60 min. Immediately transfer the mixture to an ice bath. After cooling, centrifuge at 10,000 × g for 10 min at room temperature. Transfer 200 μL supernatant into microcuvettes or a 96-well plate. Measure absorbance at 532 nm and 600 nm using a microplate reader/spectrophotometer.

GSH detection:Collect 5 million cells into 1 mL GSH working solution(Solarbio, BC1175,Beijing) and place the mixture in an ice bath. Lyse cells by ultrasonication (parameters: 200 W power, 3-sec pulses, 10-sec intervals, 30 cycles). Centrifuge at 12,000 *g for 10 min at 4°C. Carefully transfer the supernatant to fresh tubes and keep on ice pending subsequent assays. After pre-warming the microplate reader for ≥30 minutes, set the wavelength to 412 nm and measure absorbance.

### Xenograft assay

Female BALB/c nude mice (4-5 weeks, 18-22 g) sourced from the Animal Experiment Center of Yangzhou University were maintained in sterile laminar-flow strict specific pathogen-free (SPF) conditions at 25°C with 30–40% humidity under controlled 12 h light/dark cycles. Ad libitum access to standard rodent feed and water was provided. All protocols complied with ethical standards approved by Institutional Animal Care and Use Committee (IACUC) of Yangzhou University (Approval No. 202303827).Cells transduced with targeting shRNA or luciferase-tagged scramble control lentiviruses were resuspended in 100 µL PBS for subcutaneous injection into isoflurane-anesthetized (1-2%) mice flanks. Animal welfare metrics (feeding/hydration/distress behaviors) were assessed every other day. Tumor volumes calculated weekly as V = (Width²×Length)/2. Strict humane endpoints mandated euthanasia via CO_2_ asphyxiation/cervical dislocation if tumors reached 2000 mm^3^ or exhibited severe morbidity (post-mortem pupillary reflex confirmation). All mice underwent terminal procedures within 28 days for tumor extraction and weighing per Laboratory Animal Welfare Ethical Review Guidelines.

### Statistical and Survival Analysis

Quantitative results are expressed as mean ± standard deviation (SD), derived from ≥3 independent experimental replicates. Multi-group comparisons employed one-way ANOVA with Newman-Keuls post hoc analysis. Two-group comparisons utilized two-tailed Student’s t-test. Continuous variable associations were examined via Pearson correlation coefficient, while non-normally distributed or ranked variables underwent Spearman’s rank correlation analysis. Kaplan-Meier curves evaluated overall survival (OS) and disease-free survival (DFS), with group differences assessed by log-rank test. Statistical significance threshold was established at p < 0.05. All analyses were conducted using R Studio software (version 4.2.1).

## Supplementary information

Supplementary figure legends.docx

Figure legends

Supplementary Figure1

Supplementary Figure2

Supplementary Figure3

Supplementary Figure4

Supplementary Figure5

Supplementary Table 1

Supplementary Table 2

Supplementary Table 3

## Data Availability

The datasets generated and/or analysed during the current study are not publicly available due to patient privacy concerns but are available from the corresponding author on reasonable request.The transcriptome sequencing data have been uploaded to a public database.https://ngdc.cncb.ac.cn/gsa-human/browse/HRA016361.
