# Supplementary material for: Targeting the ODC1-YBX1 axis reverses gastric cancer chemoresistance via transcriptional control of SLC7A11-mediated ferroptosis
Source: Cell Death Discov. 2026 Apr 14;12:246. doi: 10.1038/s41420-026-03067-1 (PMC13194797; doi:10.1038/s41420-026-03067-1)
Supplement: Supplementary file 1 — Supplementary figure legends.docx [file 41420_2026_3067_MOESM1_ESM.docx]

Supplementary Figure S1 Multi-cohort validation of ODC1 as diagnostic and prognostic biomarker in STAD

**A)** ROC analysis of four integrated GEO training sets (n=918 samples) demnstrating ODC1's superior diagnostic performance (AUC=0.923) versus other candidates.**B)** Independent validation in three external GEO cohorts (n=112 samples) confirming ODC1's robust diagnostic accuracy (AUC=0.890).**C)** Meta-analysis of ODC1 expression across seven GEO datasets (P<0.001 for tumor vs. adjacent, paired t-test).**D-E)** Positive correlation between ODC1 expression and advanced T/N stage in TCGA-STAD .**F)** TCGA validation of ODC1 tumor overexpression.**G)** Kaplan-Meier curves showing reduced 10-year survival in high-ODC1 group (HR=1.83, P=0.0439, log-rank).*P < 0.05, **P < 0.01 , *** P < 0.001,****P<0.0001

Supplementary Figure S2 ODC1 expression in Genetically edited cell model

**A-B)** Effect of ODC1 knockdown (sh1-ODC1 and sh2-ODC1 on AGS by RT-qPCR and Western Blot assays).**C-D)**Effect of ODC1 knockdown (sh1-ODC1 and sh2-ODC1 on HGC-27 by RT-qPCR and Western Blot assays).**E-F)**Effect of ODC1 overexpression (oe-ODC1 on MKN45 by RT-qPCR and Western Blot assays). **G)** H&E and IHC staining of sh1-ODC1 and sh2-ODC1 confirms ODC1-KO efficiency and Ki-67 downregulation.*P < 0.05, **P < 0.01 , *** P < 0.001,****P<0.0001

Supplementary Figure S3 Establishment and validation of 5-Fu resistant gastric cancer models reveals ODC1-associated chemoresistance mechanisms
**A)** KEGG enrichment of differentially expressed genes in ODC1-low vs. ODC1-normal cells, showing significant involvement in drug metabolism pathways.**B)**GSEA plot confirming enrichment of xenobiotic metabolism gene set.**C)** The construction of the drug-resistant cell model(Re-AGS,Re-HGC27) was validated by calculating the IC50 through a concentration-gradient assay.**D)**Proliferation marker suppression: Western blot showing downregulation of PCNA, cyclin D1, CDK4 and CDK6 in resistant lines and parental cells.**E)** Flow cytometry demonstrating G1 arrest in resistant cells. **F)**Colony formation under 5-Fu challenge (20μM, 7days), showing increased survival in resistant lines vs parental.**G)** Calcein-AM/PI dual staining post-5-Fu, showing enhanced viable cell populations in resistant models.scale bars=20 μm.*P < 0.05, **P < 0.01 , *** P < 0.001,****P<0.0001

Supplementary Figure S4 5-Fu kills cells through ferroptosis
**A)** Western blot of cell death markers post-high-dose 5-Fu ± ODC1 depletion. **B)** ODC1 expression positively correlates with ferroptosis suppressors by TCGA correlation network. **C-D)**Calcein-AM/PI staining was performed to test cell death in ODC1-manipulated Re-AGS and Re-HGC27 cells treated with 40 μM 5-Fu for Re-HGC27,100μM 5-Fu for Re-AGS and 1 μM Fer-1, 20 μM Z-VAD, or 2 μM Nec-1 for 36 h. **E-F)**The expression of GPX4 and related ferroptosis proteins in genetically edited drug-resistant cells and parental cells was detected by immunofluorescence and Western blot. **G)**Following 5-Fu treatment, TEM analysis revealed that the drug-resistant cells underwent mitochondrial rupture. The compromised mitochondria were characterized by fewer and smaller cristae, as well as an elevated mitochondrial membrane density (labeled with white arrows).scale bars=500nm.

Supplementary Figure S5 Contextual ferroptosis dynamics and bidirectional ODC1-ferroptosis crosstalk under 5-Fu stimulation conditions

**A-B)**The expression of GPX4 and related ferroptosis proteins in sh-ODC1 drug-resistant cells with or without 5-Fu stimulation conditions was detected by immunofluorescence and Western blot.**C-D)**ROS production was measured by DCF-DA staining in sh-ODC1 drug-resistant cells with or without 5-Fu stimulation conditions.scale bars=20 μm. **E-F)** Lipid ROS generation in sh-ODC1 drug-resistant cells with or without 5-Fu stimulation conditions was measured by C11-BODIPY581/591 staining ,scale bars=20 μm.**G)** MDA levels by MDA assay kit.**H)** GSH contents by GSH assay kit. **I-J)** Iron dysregulation detected by RhoNox-6 fluorescence and Ferrous iron assay kit.scale bars=20 μm. **K)**Protein levels of ODC1 in Re-AGS or Re-HGC27 cells treated with DMSO or Erastin combined with or without DFO or Fer-1 for 24h, determined by western blotting.*P < 0.05, **P < 0.01 , *** P < 0.001,****P<0.0001
