# Supplementary figures and images for: Targeting the ODC1-YBX1 axis reverses gastric cancer chemoresistance via transcriptional control of SLC7A11-mediated ferroptosis
Source: Cell Death Discov. 2026 Apr 14;12:246. doi: 10.1038/s41420-026-03067-1 (PMC13194797; doi:10.1038/s41420-026-03067-1)

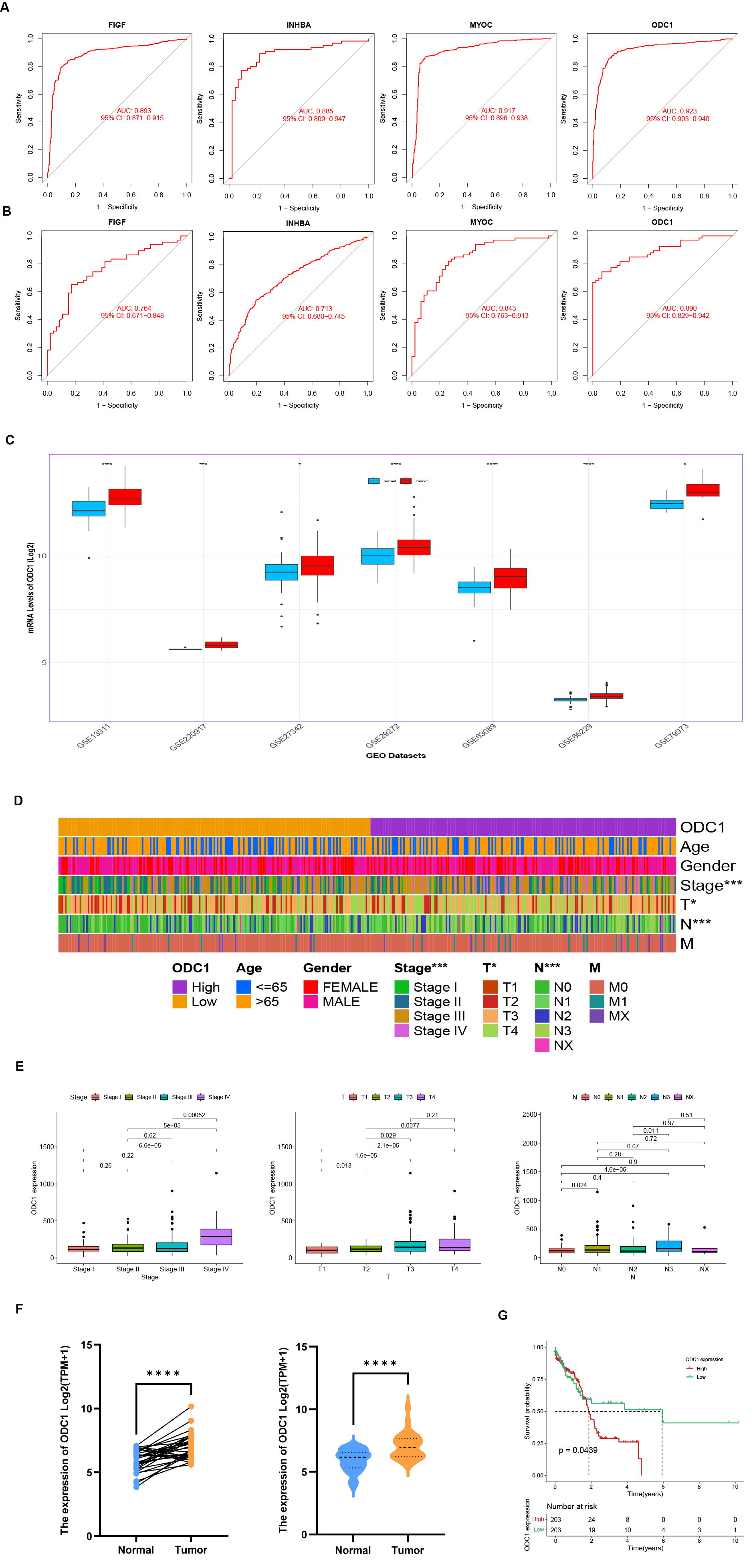

Supplement: Supplementary file 2 — Figure legends [file 41420_2026_3067_MOESM2_ESM.png]

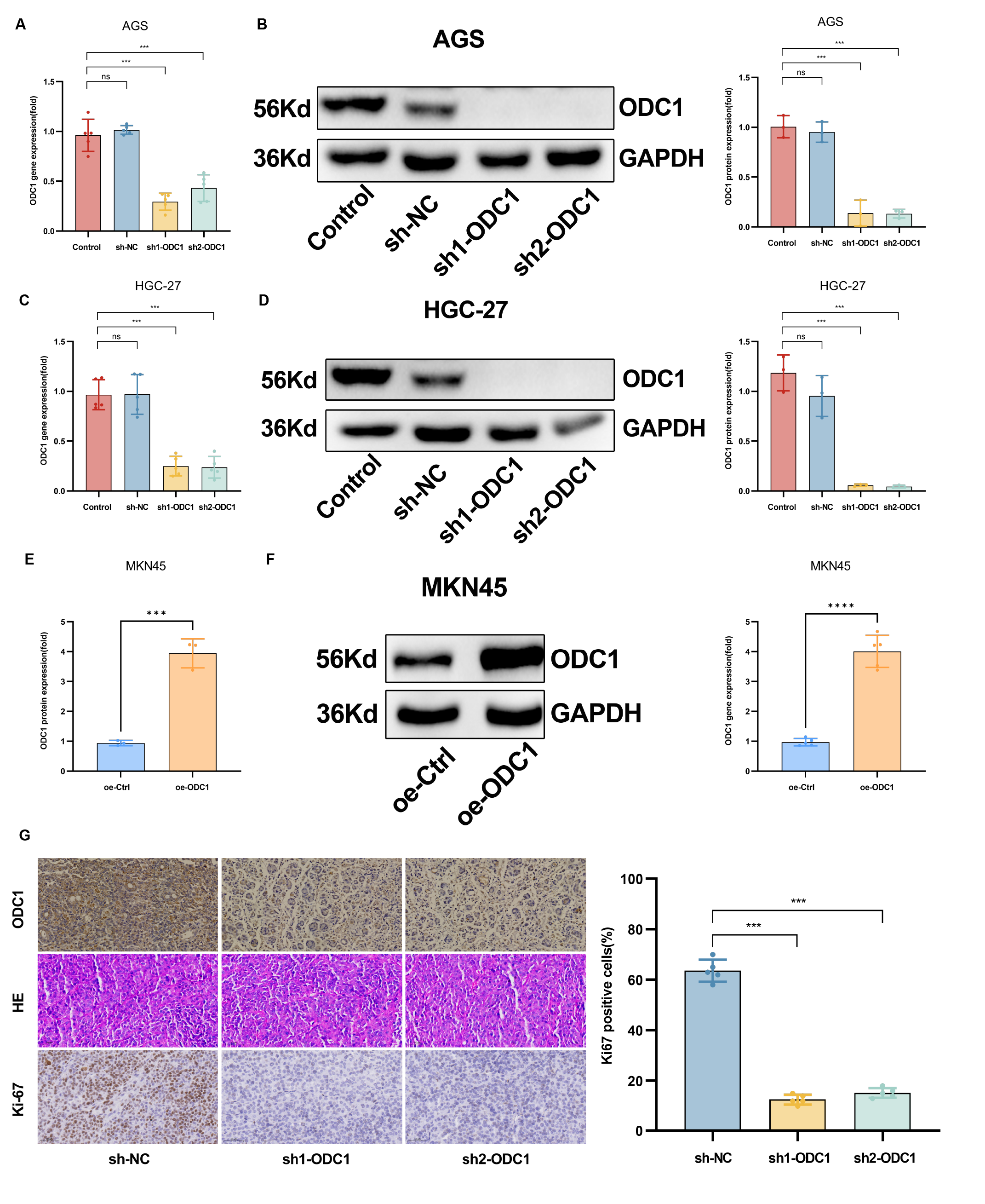

Supplement: Supplementary file 3 — Supplementary Figure1 [file 41420_2026_3067_MOESM3_ESM.png]

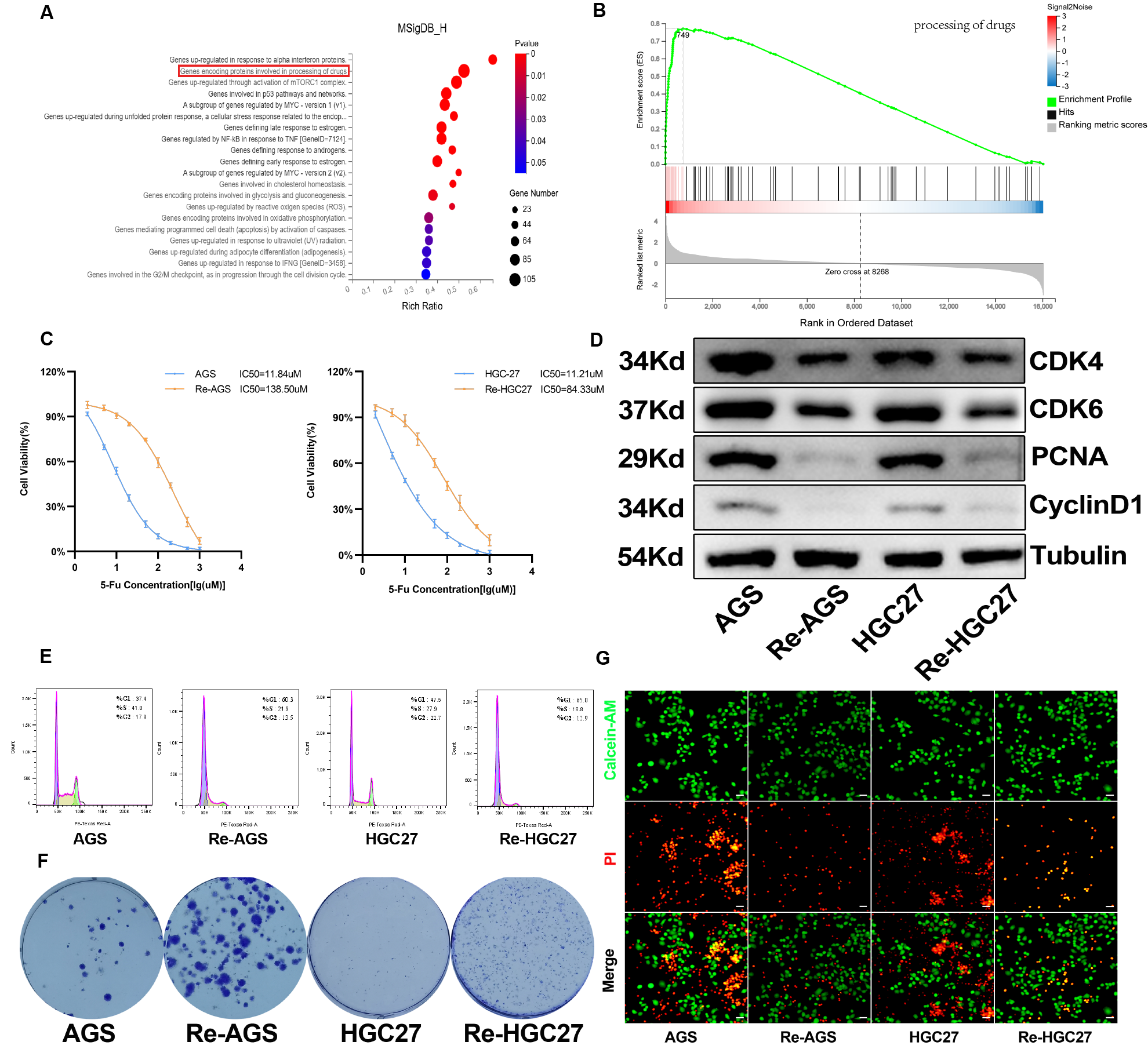

Supplement: Supplementary file 4 — Supplementary Figure2 [file 41420_2026_3067_MOESM4_ESM.png]

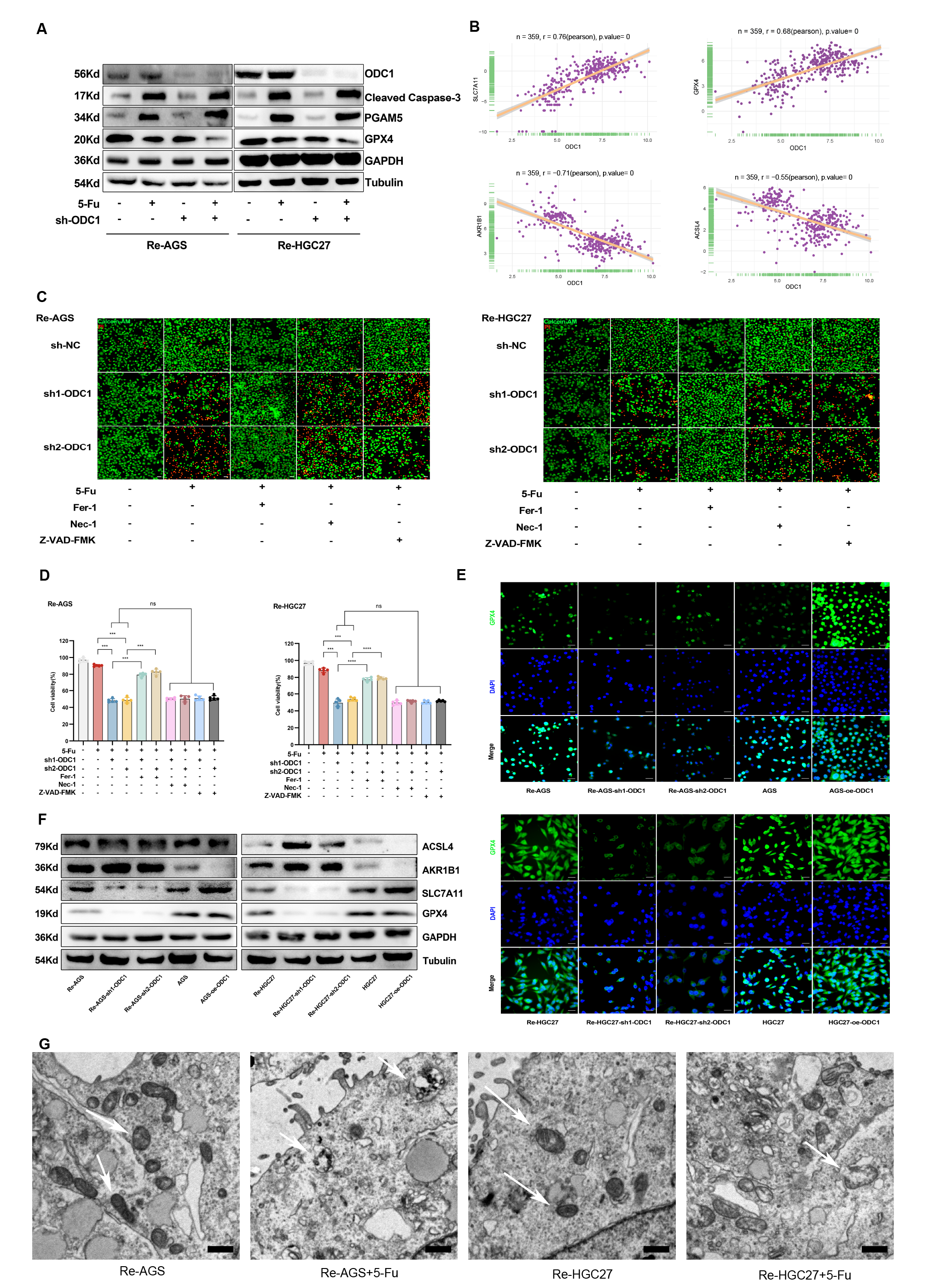

Supplement: Supplementary file 5 — Supplementary Figure3 [file 41420_2026_3067_MOESM5_ESM.png]

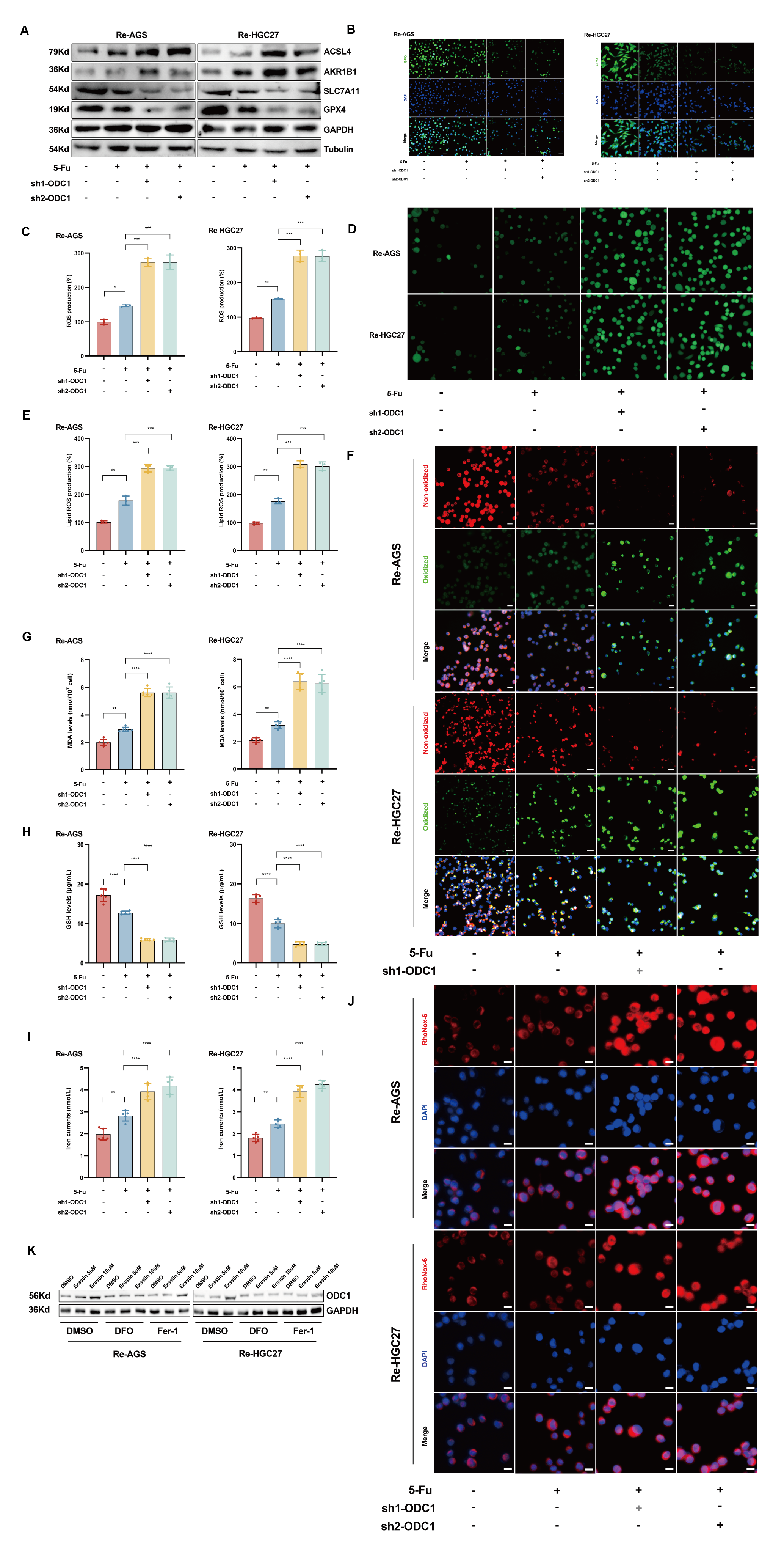

Supplement: Supplementary file 6 — Supplementary Figure4 [file 41420_2026_3067_MOESM6_ESM.png]
