# Supplementary material for: Targeting the ODC1-YBX1 axis reverses gastric cancer chemoresistance via transcriptional control of SLC7A11-mediated ferroptosis
Source: Cell Death Discov. 2026 Apr 14;12:246. doi: 10.1038/s41420-026-03067-1 (PMC13194797; doi:10.1038/s41420-026-03067-1)
Supplement: Supplementary file 7 — Supplementary Figure5 [file 41420_2026_3067_MOESM7_ESM.docx]

**Supplementary table 1. The relationship between ODC1 expression and**

**clinicopathologic features of STAD patients in our cohort**

| Variables | No. of cases (%) | High.ODC1 | Low.ODC1 | P.value |
| --- | --- | --- | --- | --- |
| All | 242(100%) | 147 | 95 |  |
| Age |  |  |  |  |
| <60 | 104(43.0%) | 56 (38.1%) | 48 (50.5%) | 0.076 |
| =>60 | 138(57.0%) | 91 (61.9%) | 47 (49.5%) |  |
| Gender |  |  |  |  |
| Female | 117(48.3%) | 76 (51.7%) | 41 (43.2%) | 0.243 |
| Male | 125(51.7%) | 71 (48.3%) | 54 (56.8%) |  |
| Sizs |  |  |  |  |
| <5cm | 179(74.0%) | 104 (70.7%) | 75 (78.9%) | 0.204 |
| =>5cm | 63(26.0%) | 43 (29.3%) | 20 (21.1%) |  |
| Lymph.node.metastasis |  |  |  |  |
| No | 125(51.7%) | 51 (34.7%) | 74 (77.9%) | 0.001 |
| Yes | 117(48.3%) | 96 (65.3%) | 21 (22.1%) |  |
| Distant.metastasis |  |  |  |  |
| No | 177(73.1%) | 99 (67.3%) | 78 (82.1%) | 0.0173 |
| Yes | 65(26.9%) | 48 (32.7%) | 17 (17.9%) |  |
| Clinical.stage |  |  |  |  |
| I + II | 156(64.5%) | 81 (55.1%) | 75 (78.9%) | 0.001 |
| III + IV | 86(35.5%) | 66 (44.9%) | 20 (21.1%) |  |
| Differentiation |  |  |  |  |
| Poor and others | 84(34.7%) | 63 (42.9%) | 21 (22.1%) | 0.00151 |
| Well and Moderate | 158(65.3%) | 84 (57.1%) | 74 (77.9%) |  |
| Status |  |  |  |  |
| Alive | 172(71.1%) | 95 (64.6%) | 77 (81.1%) | 0.00914 |
| Death | 70(28.9%) | 52 (35.4%) | 18 (18.9%) |  |
| Recurrence |  |  |  |  |
| No | 177(73.1%) | 99 (67.3%) | 78 (82.1%) | 0.0173 |
| Yes | 65(26.9%) | 48 (32.7%) | 17 (17.9%) |  |
