# Supplementary material for: Targeting the ODC1-YBX1 axis reverses gastric cancer chemoresistance via transcriptional control of SLC7A11-mediated ferroptosis
Source: Cell Death Discov. 2026 Apr 14;12:246. doi: 10.1038/s41420-026-03067-1 (PMC13194797; doi:10.1038/s41420-026-03067-1)
Supplement: Supplementary file 8 — Supplementary Table 1 [file 41420_2026_3067_MOESM8_ESM.docx]

Supplementary table 2. Primer sequences for RT-qPCR and shRNA sequences

| Name | Sequence 5’---3’ |
| --- | --- |
| ODC1 FW | TTTACTGCCAAGGACATTCTGG |
| ODC1 RW | GGAGAGCTTTTAACCACCTCAG |
| GAPDH FW | GCACCGTCAAGGCTGAGAAC |
| GAPDH RW | TGGTGAAGACGCCAGTGGA |
| ODC1 shRNA1 | ACGGGCGAAAGAGCTAAATAT |
| ODC1 shRNA2 | CTGAGGATGTGAAACTTAAAT |
| YBX1 shRNA | GGAUAUGGUUUCAUCAACATT |
