# Supplementary material for: Targeting the ODC1-YBX1 axis reverses gastric cancer chemoresistance via transcriptional control of SLC7A11-mediated ferroptosis
Source: Cell Death Discov. 2026 Apr 14;12:246. doi: 10.1038/s41420-026-03067-1 (PMC13194797; doi:10.1038/s41420-026-03067-1)
Supplement: Supplementary file 9 — Supplementary Table 2 [file 41420_2026_3067_MOESM9_ESM.docx]

Supplementary table 3. Antibodies for WB,IHC and IP

| Names | Manufacturer and catalog number | Dilution ratio |
| --- | --- | --- |
| ODC1 | Proteintect,Cat No. 28728-1-AP Wuhan,China | WB : 1:1000-1:4000  IHC : 1:200-1:800 |
| ODC1 | [Santa Cruz Biotechnology](https://www.biomart.cn/news/16/103471.htm),sc-398116,USA | IP:1-2 ug per 100-500 µg of total protein |
| GAPDH | Proteintect,Cat No. 10494-1-AP Wuhan,China | WB : 1:5000-1:40000 |
| CDK4 | Abclonal,Catalog:A23521PM  Wuhan,China | WB : 1:1000 - 1:12000 |
| CDK6 | Abclonal,Catalog:A1545  Wuhan,China | WB :1:500 - 1:1000 |
| PCNA | Abclonal,Catalog:A0264  Wuhan,China | WB :1:500 - 1:2000 |
| CyclinD1 | Abclonal,Catalog:A11022  Wuhan,China | WB :1:1000 - 1:2000 |
| Tubulin | Abclonal,Catalog:A6830  Wuhan,China | WB :1:1000 - 1:6000 |
| Cleaved-Caspase3 | Proteintect,Cat No. 25128-1-AP Wuhan,China | WB : 1:500-1:2000 |
| PGAM5 | Proteintect,Cat No. 28445-1-AP Wuhan,China | WB : 1:2000-1:14000 |
| GPX4 | Proteintect,Cat No. 30388-1-AP Wuhan,China | WB : 1:500-1:2000 |
| ACSL4 | Proteintect,Cat No. 81196-1-RR Wuhan,China | WB : 1:5000-1:50000 |
| AKR1B1 | Proteintect,Cat No. 15439-1-AP Wuhan,China | WB : 1:500-1:3000 |
| SLC7A11 | Abclonal,Catalog:A25302  Wuhan,China | WB :1:500 - 1:1000 |
| YBX1 | Abclonal,Catalog:A3534  Wuhan,China | WB :1:1000 - 1:6000 |
| Ki-67 | Abclonal,Catalog:A20018  Wuhan,China | WB :1:2000 - 1:10000 |
| IgG | Proteintect,Cat No. B900620  Wuhan,China | IP:1-2 ug per 100-500 µg of total protein |
| Flag | Proteintect,Cat No. 66008-4-Ig  Wuhan,China | IP : 0.5-4.0 ug for 1.0-3.0 mg of total protein lysate |
| Rabbit secondary antibody | Proteintect,Cat no : SA00001-2 Wuhan,China | WB :1:2,000-1:10,000 |
| Mouse secondary antibody | Proteintect,Cat No. 80015-1-RR Wuhan,China | WB : 1:5000-1:50000 |
