# Supplementary material for: Targeting the ODC1-YBX1 axis reverses gastric cancer chemoresistance via transcriptional control of SLC7A11-mediated ferroptosis
Source: Cell Death Discov. 2026 Apr 14;12:246. doi: 10.1038/s41420-026-03067-1 (PMC13194797; doi:10.1038/s41420-026-03067-1)

Original western blots

Fig. 2I-ODC1

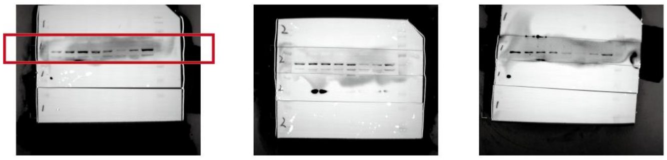

Fig. 2I-GAPDH

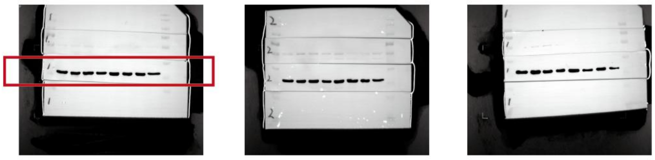

Fig. 4B-ODC1

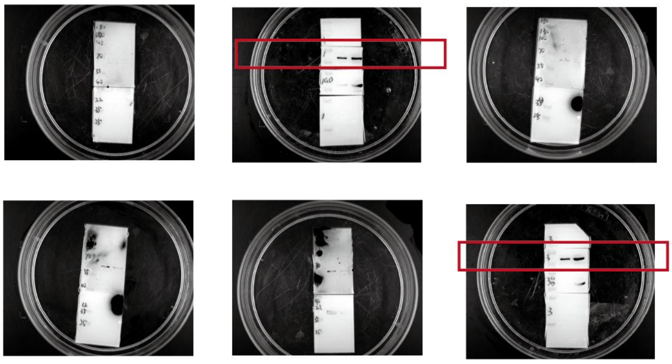

Fig. 4B-GAPDH

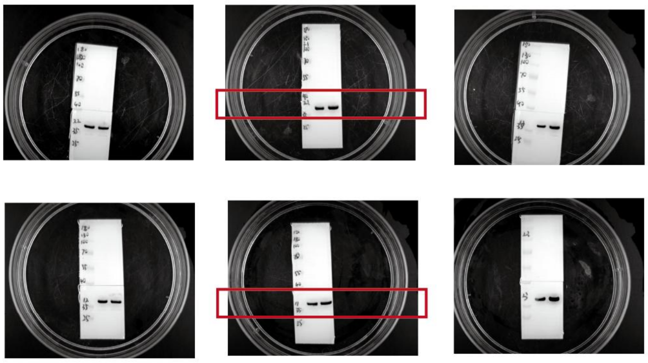

Fig. 4C-shODC1-Re-AGS and Re-HGC27

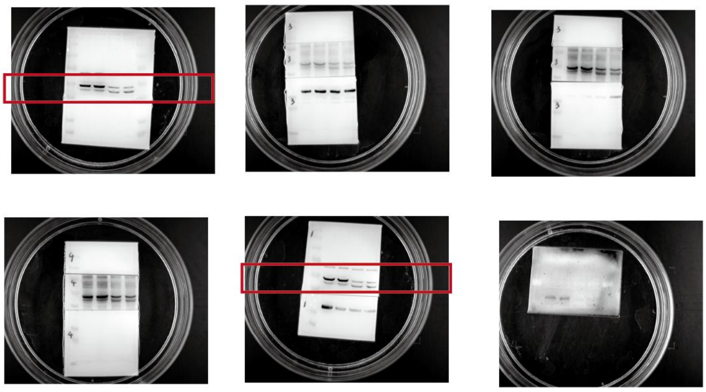

Fig. 4C-GAPDH--Re-AGS and Re-HGC27

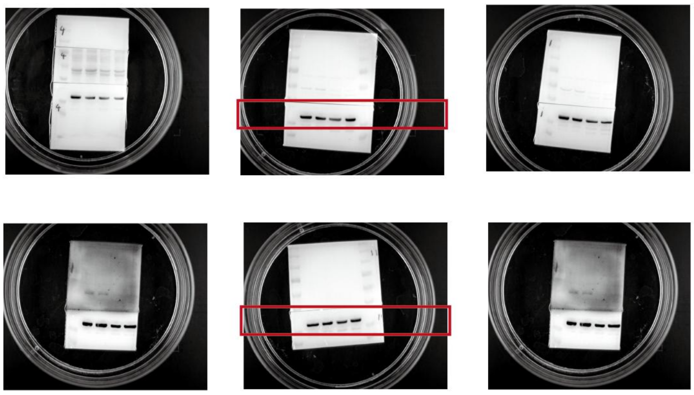

Fig. 4C-oeODC1-AGS and HGC27

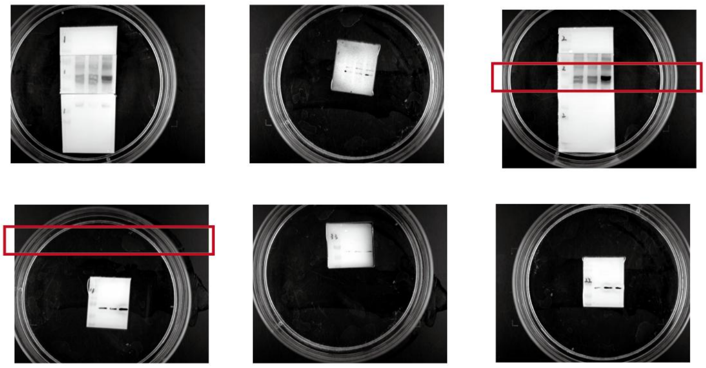

Fig. 4C-GAPDH--AGS and HGC27

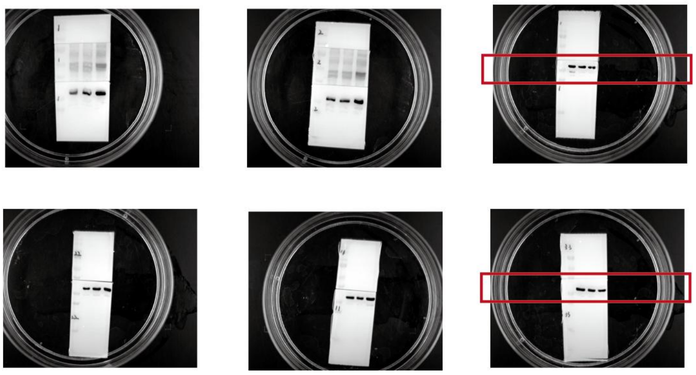

Original western blots

Fig. 4E-CDK4-sh-Re-AGS and Re-HGC27

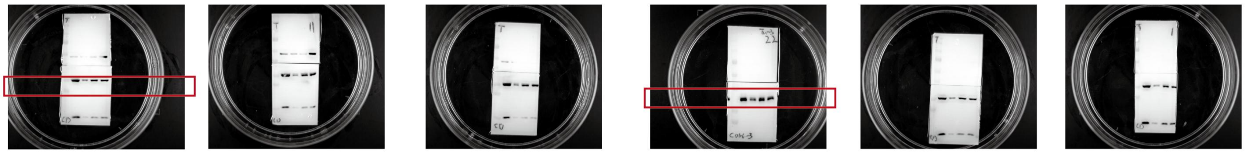

Fig. 4E-CDK4-oe-Re-AGS and Re-HGC27

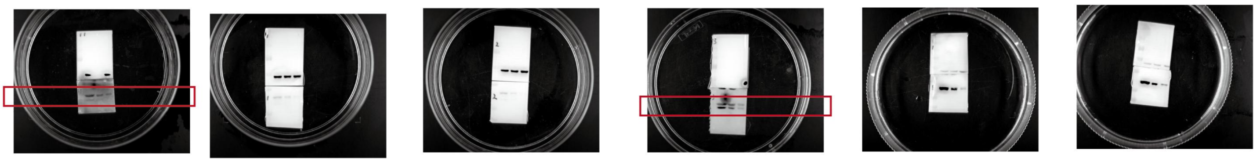

Fig. 4E-CDK6-sh-Re-AGS and Re-HGC27

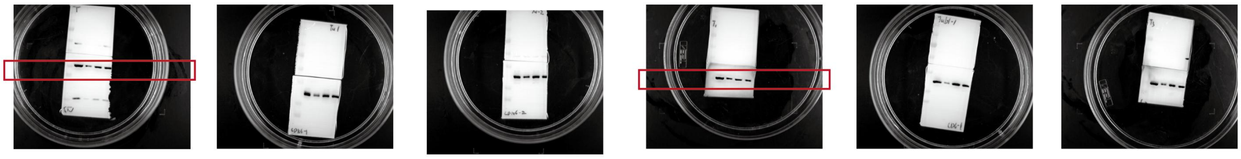

Fig. 4E-CDK6-oe-Re-AGS and Re-HGC27

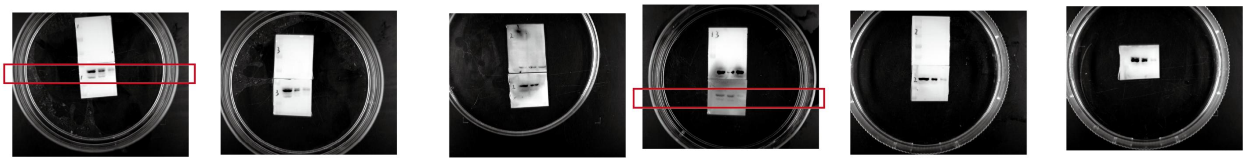

Fig. 4E-PCNA-sh-Re-AGS and Re-HGC27

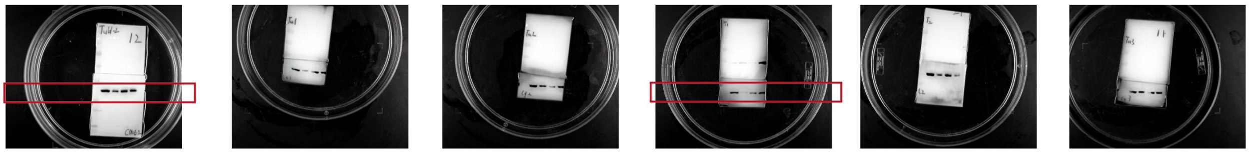

Fig. 4E-PCNA-oe-Re-AGS and Re-HGC27

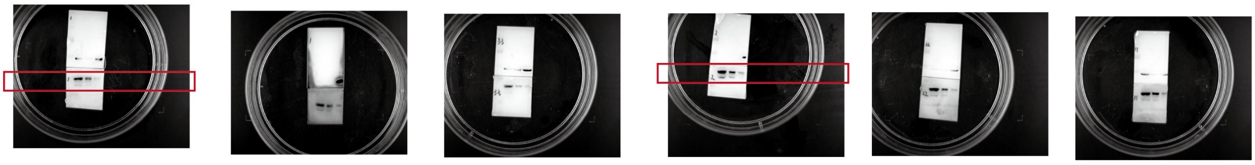

Fig. 4E-CyclinD1-sh-Re-AGS and Re-HGC27

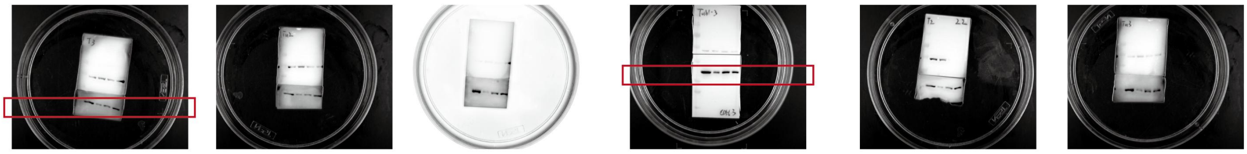

Fig. 4E-CyclinD1-oe-Re-AGS and Re-HGC27

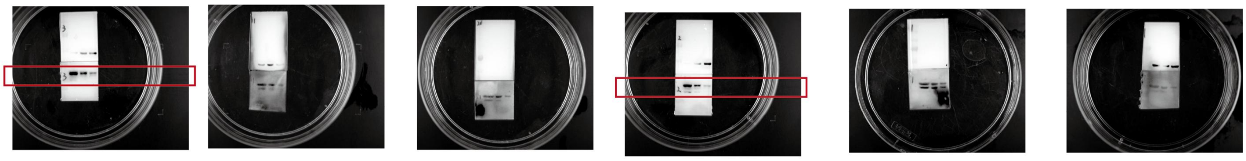

Fig. 4E-Tubulin-sh-Re-AGS and Re-HGC27

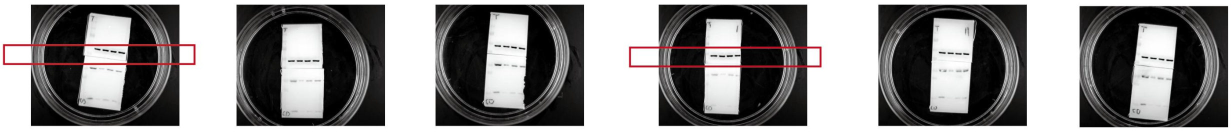

Fig. 4E-Tubulin-oe-Re-AGS and Re-HGC27

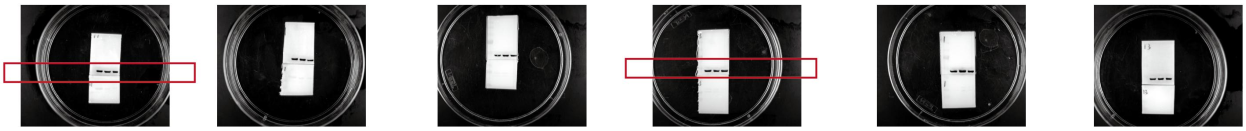

Original western blots

Fig. 5A-ODC1

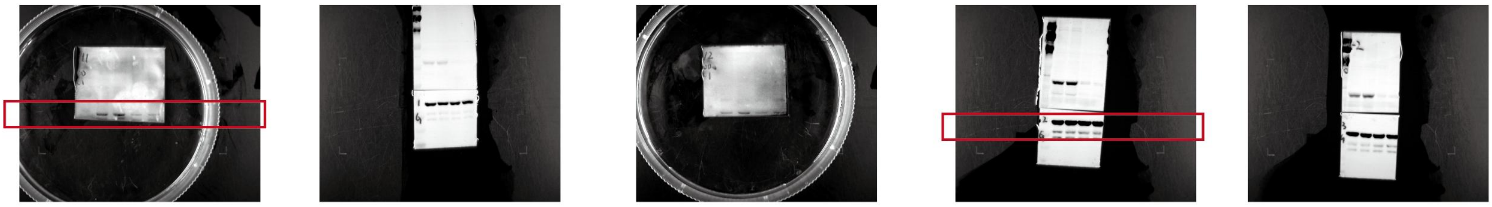

Fig. 5A-cleaved-caspase3

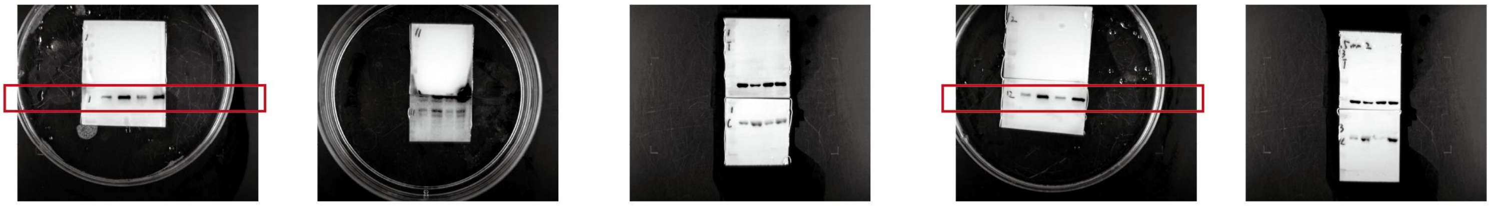

Fig. 5A-PGAM5

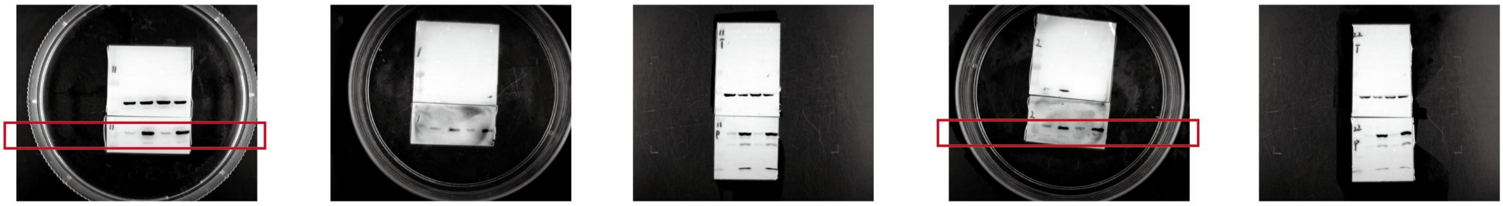

Fig. 5A-GPX4

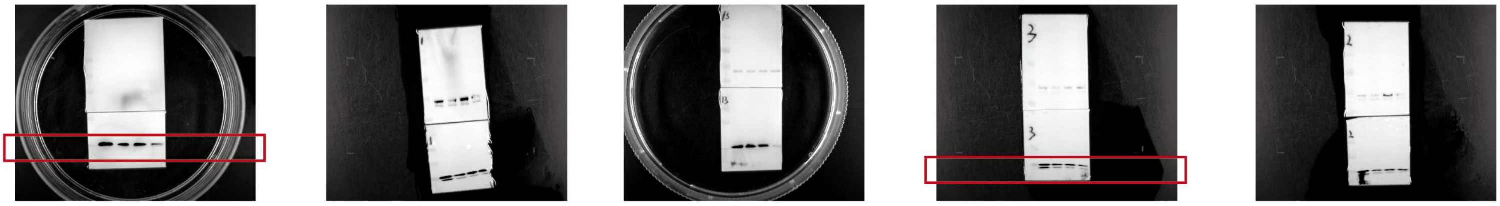

Fig. 5A-GAPDH

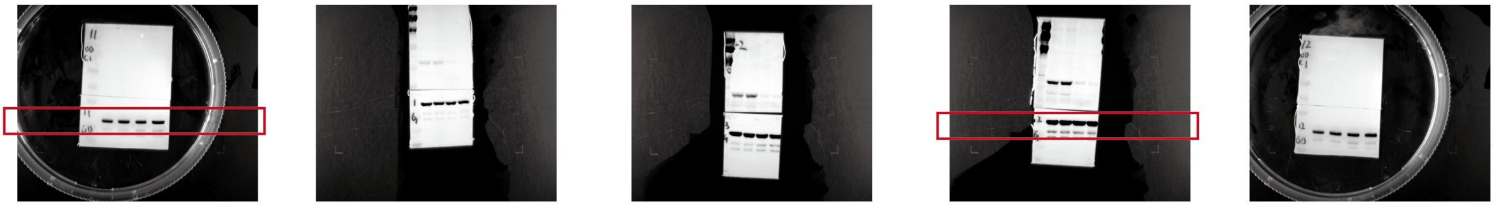

Fig. 5A-Tubulin

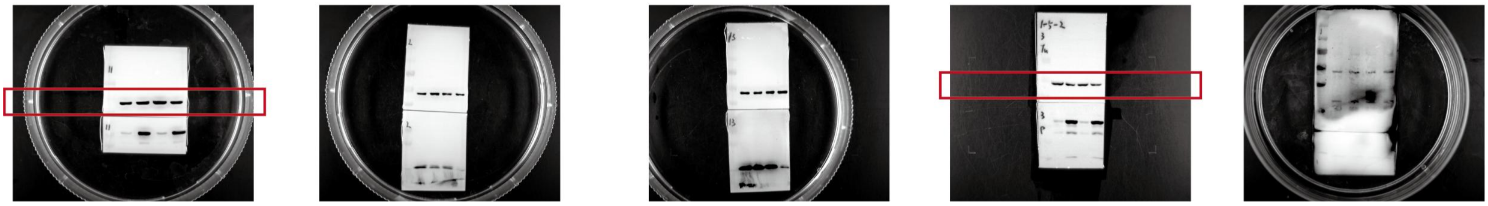

Original western blots

Fig. 5F-ACSL4

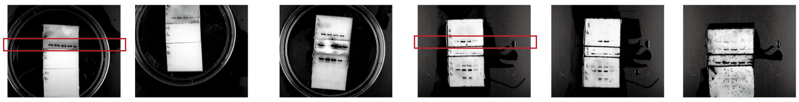

Fig. 5F-AKR1B1

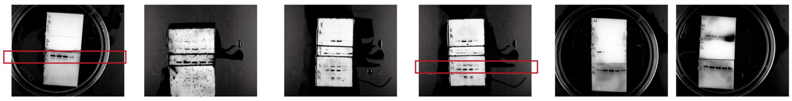

Fig. 5F-SLC7A11

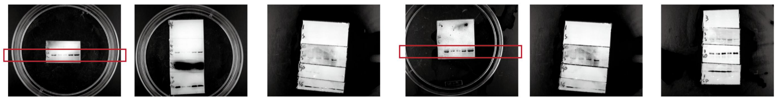

Fig. 5F-GPX4

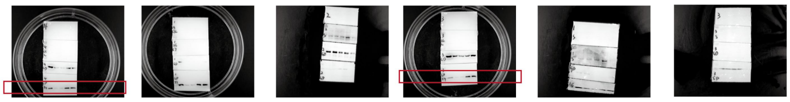

Fig. 5F-GAPDH

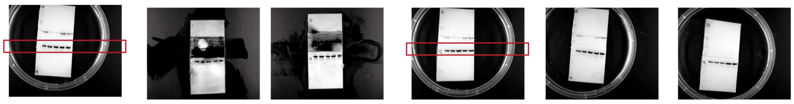

Fig. 5F-Tubulin

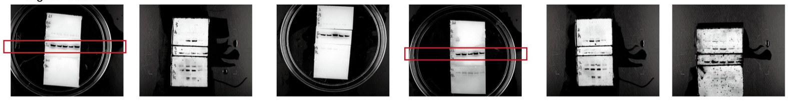

Fig. 7O-ACSL4 -ReAGS and ReHGC27

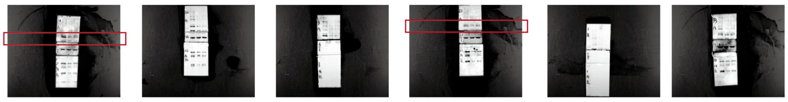

Fig. 7O-AKR1B1 -ReAGS and ReHGC27

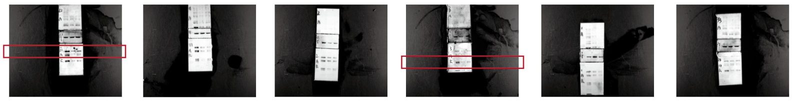

Fig. 7O-GPX4 -ReAGS and ReHGC27

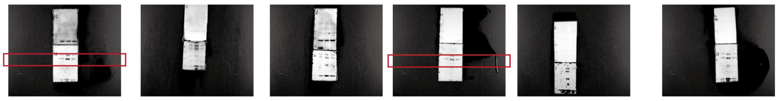

Fig. 7O-Tubulin -ReAGS and ReHGC27

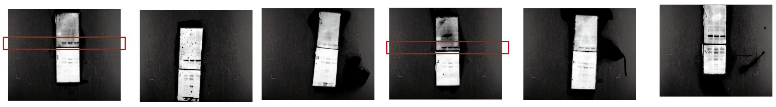

Fig. 7P-ACSL4 -ReAGS and ReHGC27

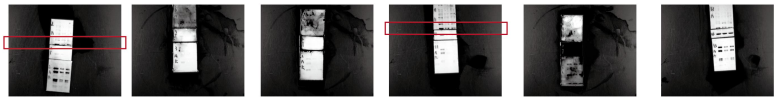

Fig. 7P-AKR1B1 -ReAGS and ReHGC27

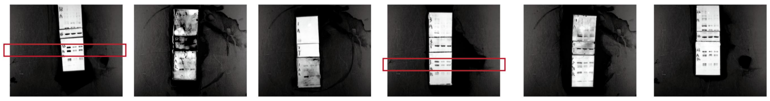

Fig. 7P-GPX4 -ReAGS and ReHGC27

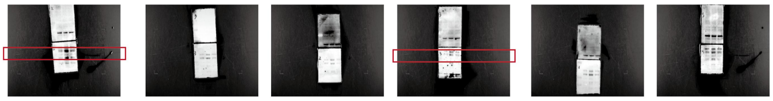

Fig. 7P-Tubulin -ReAGS and ReHGC27

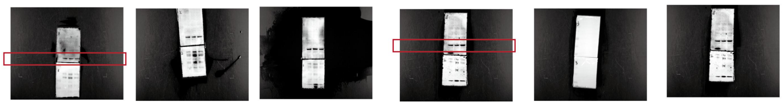

Original western blots

Fig. 8A-SLC7A11

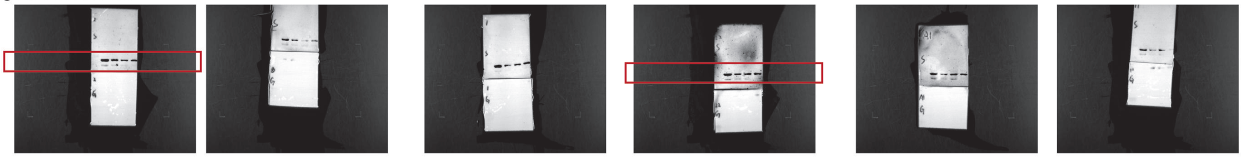

Fig. 8A-GAPDH

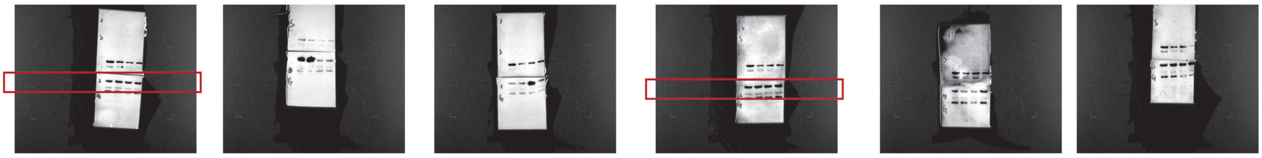

Supplementary Figure 2B - ODC1

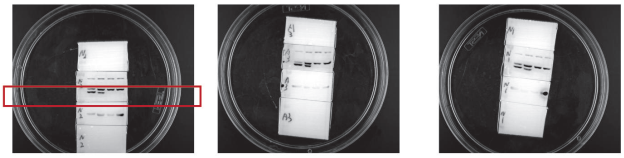

Supplementary Figure 2B - GAPDH

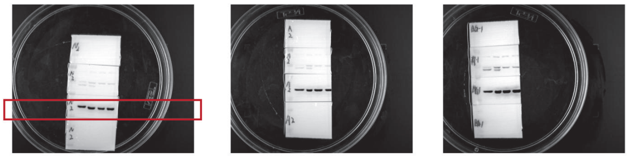

Supplementary Figure 2D - ODC1

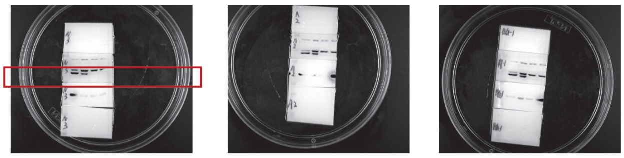

Supplementary Figure 2D - GAPDH

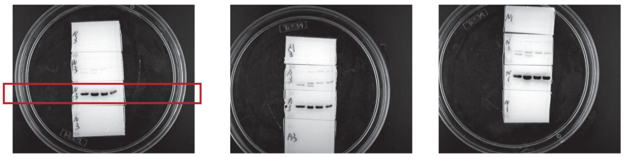

Supplementary Figure 2F - ODC1

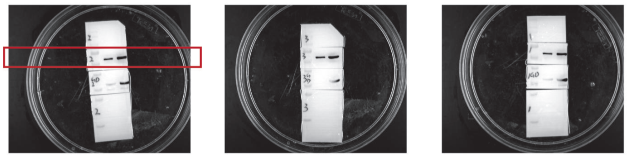

Supplementary Figure 2F - GAPDH

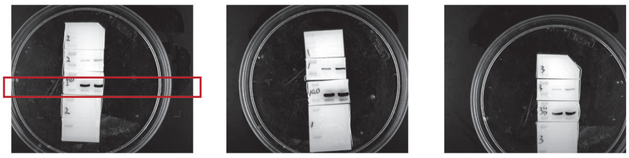

Supplementary Figure 3D - CDK4

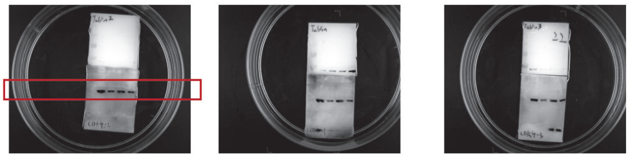

Supplementary Figure 3D - CDK6

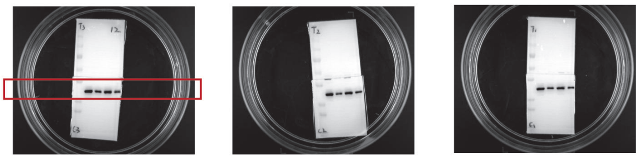

Supplementary Figure 3D - PCNA

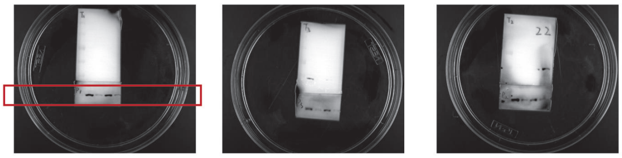

Supplementary Figure 3D - CyclinD1

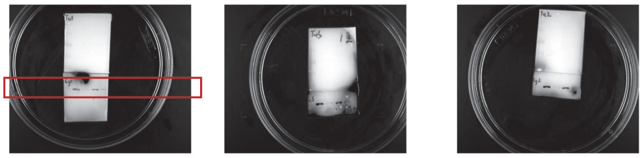

Supplementary Figure 3D - Tubulin

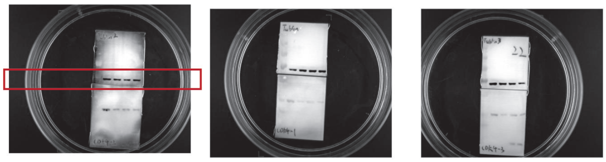

Supplementary Figure 4K - ODC1

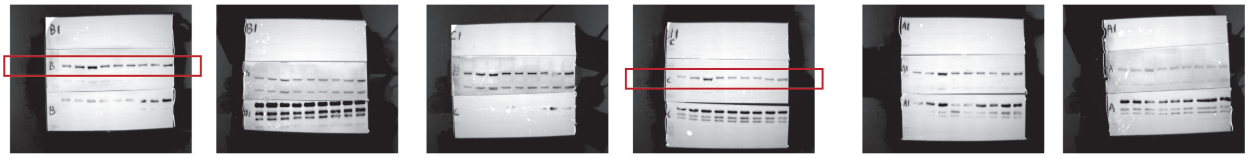

Supplementary Figure 4K - GAPDH

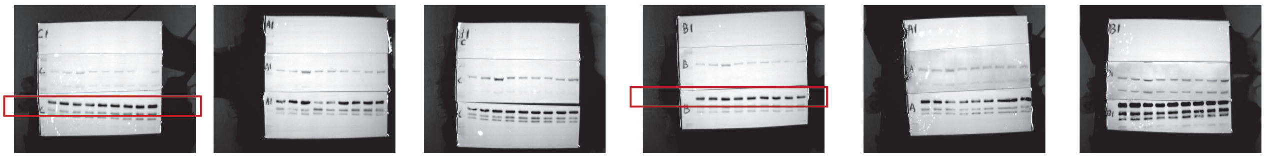

Original western blots

Fig. 6G-SLC7A11

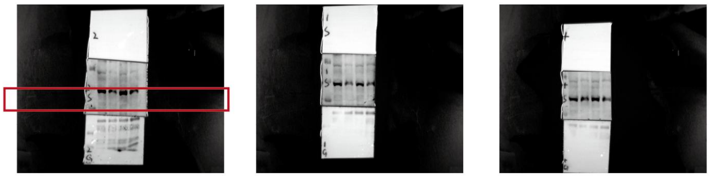

Fig. 6G-GAPDH

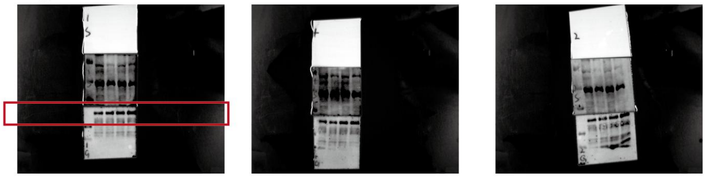

Fig. 6G-YBX1

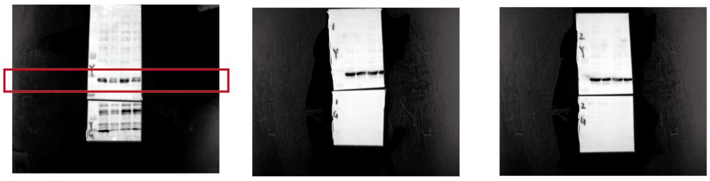

Fig. 6N-SLC7A11

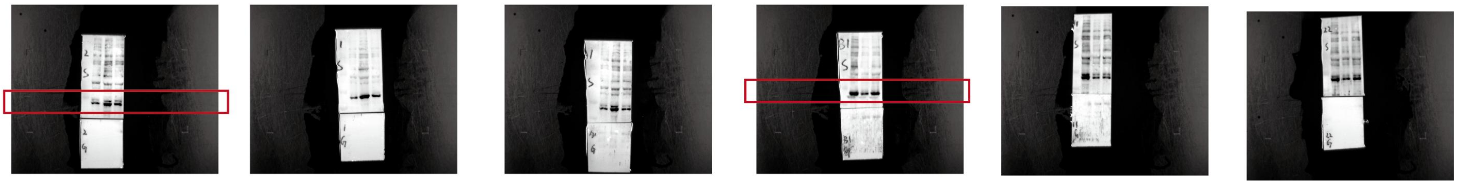

Fig. 6N-GAPDH

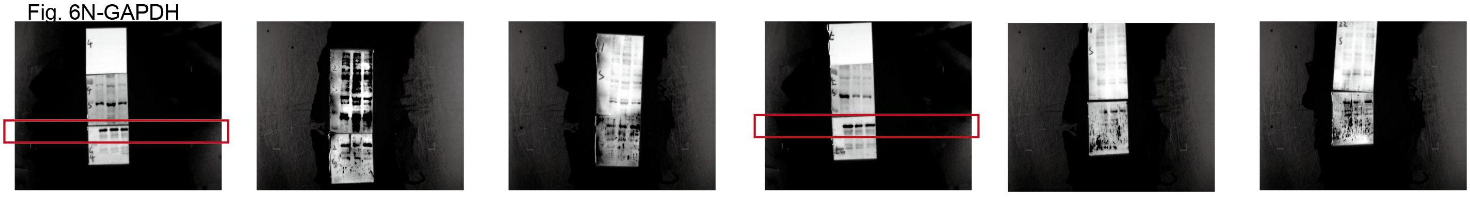

Supplementary Fig4-ACSL4

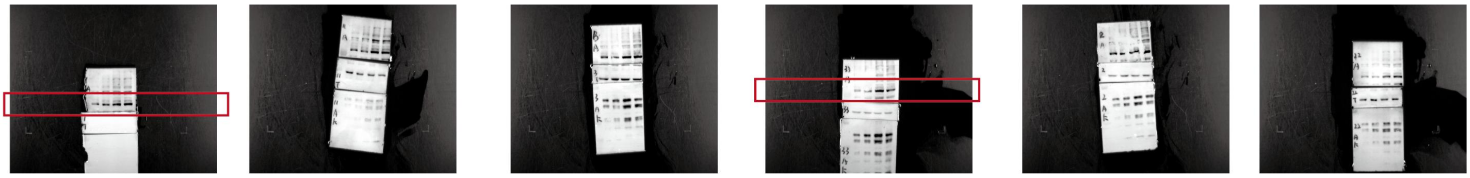

Supplementary Fig4-AKR1B1

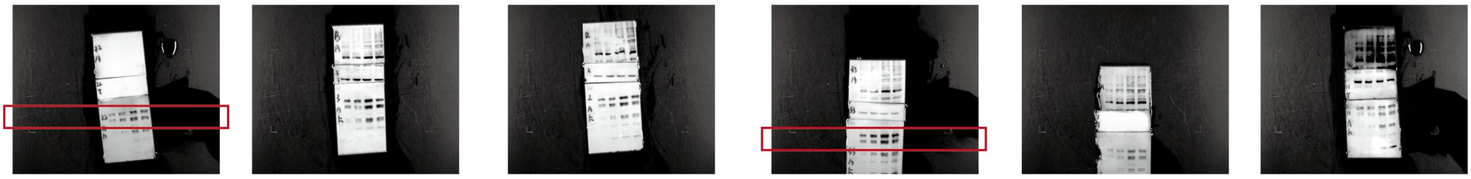

Supplementary Fig4-SLC7A11

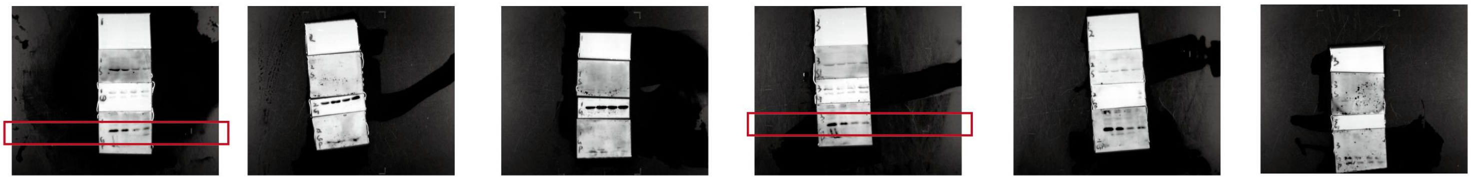

Supplementary Fig4-GAPDH

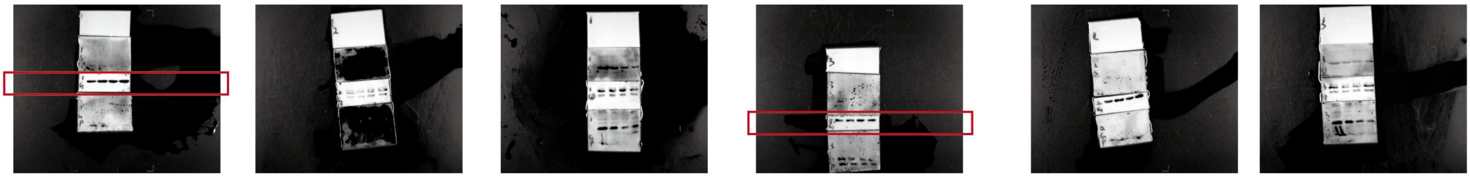

Supplementary Fig4-Tubulin

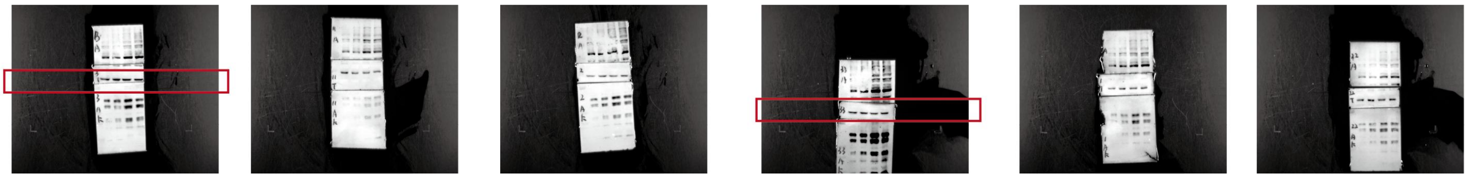

Supplement: Supplementary file 10 — Supplementary Table 3 [file 41420_2026_3067_MOESM10_ESM.pdf]
